# Idiopathic Pulmonary Fibrosis: Cellular Heterogeneity, Mechanisms, and Therapeutic Implications

**DOI:** 10.1002/mco2.70521

**Published:** 2025-12-15

**Authors:** Lin Zuo, Qiongliang Liu, Defeng Ye, Jiang Fan, Liang Wu

**Affiliations:** ^1^ Department of Thoracic Surgery Shanghai General Hospital, Shanghai Jiao Tong University School of Medicine Shanghai China

**Keywords:** epithelial cells, fibroblasts, immune cells, IPF, scRNA‐seq, spatial transcriptomics

## Abstract

Idiopathic pulmonary fibrosis (IPF) is a progressive and fatal interstitial lung disease characterized by excessive extracellular matrix (ECM) deposition and irreversible alveolar destruction. Despite advances in antifibrotic therapies, the underlying pathogenic mechanisms remain incompletely understood. Recent multiomic studies have revealed that IPF arises from aberrant communication among epithelial, mesenchymal, immune, and vascular cells within the fibrotic microenvironment, rather than from isolated cellular dysfunction. However, the dynamic intercellular networks and spatiotemporal regulation driving disease progression remain poorly defined. This review integrates recent single‐cell RNA sequencing and spatial transcriptomic discoveries to delineate key pathogenic cell populations—including aberrant basaloids and IPF‐related alveolar type 2 cells (IR_AT2), CTHRC1^+^ and meflin^+^ fibroblasts, and SPP1^hi^ macrophages—and their signaling crosstalk through pathways such as transforming growth factor β(TGF‐β), Hippo, and Hedgehog. We further discuss how ECM feedback loops and immune‐metabolic remodeling reinforce fibrogenesis and explore emerging therapeutic targets derived from these mechanisms. By synthesizing multidimensional data into a cellular and molecular framework, this review advances the understanding of IPF pathogenesis and provides a conceptual foundation for biomarker‐guided precision therapies.

## Graphical abstract

Single‐cell sequencing has elucidated cell‐type‐specific transcriptional alterations, activated signaling pathways, metabolic reprogramming, immune infiltration, and chromatin‐associated genetic mechanisms in IPF, uncovering novel therapeutic targets. This approach has identified dynamic changes in cellular biomarkers and unrecognized molecular signatures during IPF progression. Spatial transcriptomics has further contextualized these molecular changes by mapping their spatial distribution within fibrotic lesions.

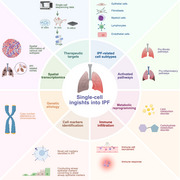

## 1 Introduction

IPF is a progressive, fatal interstitial lung disease characterized by the replacement of healthy lung tissue with abnormal ECM, leading to impaired lung structural integrity and gas exchange. This pathological remodeling results in respiratory failure and often culminates in death [1]. The progression of IPF presents with worsening cough and dyspnea, significantly decreasing patients’ quality of life. This disease affects an estimated 3 million individuals globally [2], with a median survival time of 3–5 years after diagnosis [3]. Risk factors for IPF include environmental exposure, such as metal dust, wood dust, pesticides, agricultural activities, and smoking [4]. While the precise causes of IPF remain unclear, its pathogenesis is widely accepted to involve a complex interplay of various cellular and molecular mechanisms, including repeated epithelial injury in the lungs, aberrant fibroblast activation, and excessive ECM deposition [5, 6].

Over the past decade, the use of antifibrotic agents, such as pirfenidone and nintedanib, has become the standard of care and has led to a demonstrably slow decline in lung function [7]; however, these agents do not halt disease progression, and their impact on long‐term survival remains limited. Recent large trials of next‐generation agents, such as the anticonnective tissue growth factor (anti‐CTGF) antibody pamrevlumab in ZEPHYRUS‐1 [8] and the pentraxin‐2 analog zinpentraxin alfa in STARSCAPE [9], have failed to meet the primary endpoints, underscoring the need for new therapeutic strategies.

**FIGURE 1:**
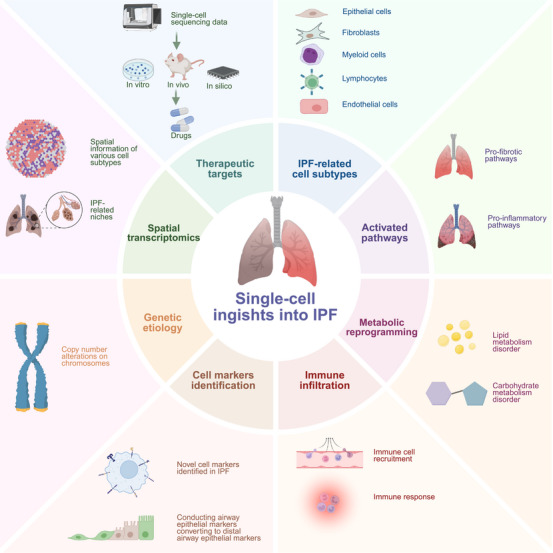
Overview of single‐cell insight into IPF. Single‐cell sequencing provide multifaceted perspectives on IPF pathogenesis and identifies novel therapeutic targets. This cutting‐edge approach has delineated cell‐type‐specific transcriptional alterations across pulmonary cell populations, attributing to identify activated signaling pathways, metabolic reprogramming events, immune infiltration patterns, and chromatin‐associated genetic etiology. Furthermore, single‐cell analyses have revealed dynamic changes in cellular biomarkers during IPF development, leading to the discovery of previously unrecognized molecular signature. Spatial transcriptomics has complemented these findings by mapping the spatial distribution of transcriptional alterations within fibrotic lesions, providing crucial anatomical context for observed molecular changes. *Abbreviations*: IPF, idiopathic pulmonary fibrosis; EMT, epithelial–mesenchymal transition.

Mechanistic insights obtained over the past two decades have shifted the understanding of IPF from a purely inflammation‐driven disease to one centered on repetitive alveolar epithelial injury, aberrant repair, and persistent fibroblast–myofibroblast activation driven by profibrotic mediators, most notably TGF‐β [6]. Given this evolving background, advances in single‐cell RNA sequencing (scRNA‐seq) and spatial transcriptomics have provided unprecedented resolution of the cellular and microenvironmental architecture of the fibrotic lung. These technologies have revealed novel cell populations, such as “aberrant basaloid” epithelial cells at the edge of fibroblastic foci [10], and their spatial organization alongside fibroblast–myofibroblast lineages [11] and ectopic endothelial cells [12]. Spatial multiomics approaches now allow these cell states to be mapped to specific anatomical contexts, identifying fibroblast–epithelium–immune interaction hubs that may drive lesion expansion [13]. This shift not only refines our mechanistic understanding but also opens avenues for biomarker‐guided interventions that target this disease at the level of cellular neighborhoods rather than the whole organ (Figure 1).

In this review, we first summarize the latest single‐cell and spatial transcriptomic insights into IPF, focusing on epithelial, mesenchymal, and immune cell states and their spatial interactions. Second, we explore how these discoveries inform the development of novel therapeutic strategies and personalized treatment approaches. Finally, we discuss the challenges and future perspectives of single‐cell research on IPF pathogenesis.

## 2 The IPF Ecosystem: Cellular Players and Their Roles in Pathogenesis

IPF does not arise from abnormalities in a single cell type but from a spatially organized network of cell–cell and cell–matrix interactions that converts recurrent epithelial injury into persistent fibrogenesis. In pathogenic niches—most prominently at the edge of fibroblastic foci and along distal airways—maladaptive alveolar epithelial states catalyze the activation of several molecular pathways such as TGF‐β signaling pathway, which licenses fibroblast lineage expansion and myofibroblast differentiation. Concomitantly, immune and inflammatory programs remodel the milieu by delivering profibrotic cues and metabolic signals, while ECM deposition increases tissue stiffness, reinforcing mechano‐transduction (e.g., Yes‐associated protein [YAP]/TAZ) and further amplifying ECM production. These axes interlock into self‐sustaining, feed‐forward loops that couple epithelial stress, immune regulation, fibroblast heterogeneity, and biomechanics into a progressive disease trajectory.

### 2.1 The Initiators: Lung Epithelial Cell Injury and Dysfunction

Epithelial cell injury and dysfunction plays a critical role in the pathogenesis of IPF [14, 15]. Recent studies have shifted the focus from viewing IPF primarily as an inflammatory disease to understanding it as a condition driven by recurrent microinjury to the alveolar epithelium, leading to fibrotic remodeling [5, 14]. This shift suggests that epithelial cell injury and dysfunction play a pivotal role as the switch in the pathogenesis of IPF.

#### 2.1.1 Altered Expression Patterns of Epithelial Cells in IPF

**FIGURE 2:**
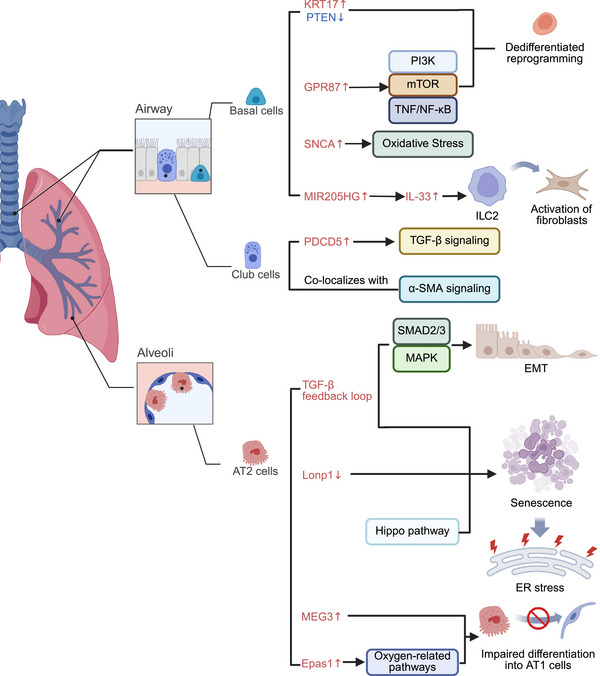
Reprogramming of epithelial cells in IPF. In IPF pathogenesis, airway basal cells exhibit a dedifferentiated phenotype characterized by elevated KRT17 expression and reduced PTEN levels. Upregulation of GPR87 in basal cells promotes dedifferentiation reprogramming through the PI3K/mTOR and TNF/NF‐κB pathways, while *SNCA* contributes to oxidative stress. MIR205HG enhances ILC2‐mediated fibroblast activation by inducing IL‐33 overexpression. Club cells in IPF colocalize with α‐SMA signals and highly express PDCD5, which facilitates TGF‐β‐induced fibrotic responses. AT2 cells demonstrate impaired differentiation into AT1 cells due to upregulated *Epas1* and MEG3. Downregulation of *Lonp1*, activation of the TGF‐β feedback loop, and dysregulated Hippo signaling collectively drive AT2 cell senescence, exacerbating fibrosis via ER stress. Furthermore, the TGF‐β feedback loop induces EMT through SMAD2/3 and MAPK signaling pathways. *Abbreviations*: ILC2, type 2 innate lymphoid cells; α‐SMA, α‐smooth muscle actin; EMT, epithelial–mesenchymal transition; ER stress, endoplasmic reticulum stress.

In the initiation phase of IPF, alveolar epithelial cells act as the trigger for disease onset, undergoing numerous molecular alterations. Identifying these changes could help in discovering early therapeutic targets for disease intervention. Recent advances in transcriptomics and epigenetic profiling have revealed profound changes in the gene expression patterns of epithelial cells in IPF lungs (Figure 2). Numerous studies have shown that in IPF, peripheral lung epithelial cells lose their normal alveolar epithelial gene expression patterns and instead express genes associated with various conducting airway epithelial cells [10, 16, 17, 18]. Moreover, genes involved in early lung morphogenesis and the Wnt signaling pathway are highly expressed in IPF epithelial cells but are barely expressed in normal AT2 cells. Additionally, MEG3, a long noncoding RNA (lncRNA) associated with the conducting airway epithelial gene expression pattern, is rarely detected in normal lung epithelial cells but is highly expressed in atypical epithelial cell subpopulations in IPF [16]. MEG3 binds to the promoter regions of genes that regulate airway epithelial cell differentiation in epithelial cells and upregulates the expression of basal cell‐associated RNAs while increasing cell migration. On the other hand, MEG3 downregulates the expression of TP73, SOX2, and Notch‐related RNAs in primary human bronchial epithelial cells, highlighting its role in inhibiting genes that affect basal cell differentiation into club cells, ciliated cells, or goblet cells. MEG3 induces the expression of basal cell genes while suppressing genes associated with the terminal differentiation of airway cells, which may contribute to tissue remodeling in IPF [16]. Accordingly, these transcriptional changes in epithelial cells align with the characteristic “bronchiolization” lesions commonly seen in IPF lung tissue.

In IPF, epithelial cells also exhibit significant dysregulation of ubiquitination [19]. In lung injury, ubiquitination is secondary to the inflammatory response, influencing the barrier function of alveolar epithelial cells by targeting sodium channels in the damaged lung [20]. Wen and Ablimit [19] screened hub genes correlated with ubiquitination abnormalities in IPF epithelial cells and found that ubiquitin‐ribosomal protein eL40 fusion protein, polyubiquitin‐C, and polyubiquitin‐B are strongly correlated with cell matrix‐related genes, indicating that protein ubiquitination might contribute to tissue fibrosis.

Recently, studies have investigated the epithelial cell‐specific genomic basis of IPF through a combination of single‐cell sequencing with inferCNV technology. Specifically, researchers identified an increase in copy number alteration events on chromosome 16 and a decrease on chromosome 19, providing insights into the potential genetic etiology of the disease [21].

Taken together, these findings underscore the multifaceted nature of epithelial cell dysfunction in IPF, involving transcriptional reprogramming, posttranslational modifications, and genomic instability. These epithelial‐specific alterations provide potential molecular targets, such as lncRNAs, ubiquitination pathways, and chromosomal aberrations, for future therapeutic interventions.

#### 2.1.2 Functions and Heterogeneity of AT2 Cells in IPF

AT2 cells are essential stem cells that maintain the lung epithelium in distal gas exchange regions and play crucial roles in homeostatic turnover and the response to injury through their differentiation into AT1 cells [22]. The dysfunction of AT2 cells plays a crucial role in the pathogenesis of IPF, which leads to the activation of fibroblasts, disruption of the lung architecture, and excessive deposition of ECM, ultimately contributing to the development of pulmonary fibrosis [23, 24, 25]. The main characteristic of AT2 cells in the pathogenesis of IPF is impaired differentiation into AT1 cells or other cell types, with the inability to repair the damaged epithelium [10]. Recent studies have highlighted the distinct expression profiles of AT2 cells during this impaired differentiation process. In a bleomycin (BLM)‐induced murine model of lung fibrosis, the expression of several fibrosis‐related genes peaked in parallel with the impaired differentiation of AT2 cells [26].

Additionally, several transitional AT2 cells and aberrant AT2 cell subpopulations resulting from differentiation defects have been identified and characterized (Table 1). KRT5^−^/KRT17^+^ epithelial cells are characterized by their close association with lesions with high levels of collagen deposition in distal regions of the fibrotic lung. Pseudotime analysis indicated that these cells originate from AT2 cells [12]. Reanalysis of multiple scRNA‐seq datasets further confirmed that this epithelial cell subtype represents a transitional cell state with activated SMAD3 and Notch signaling, which plays a role in differentiation from AT2 cells [18], indicating that the AT2 cell lineage is involved in the initiation of fibrosis. KRT5^−^/KRT17^+^ epithelial cells are enriched in fibrosis‐related genes such as COL1A1, FN1, CDH2, and other pathological ECM components [12]. Moreover, these cells exhibit a gene expression profile similar to that of the aberrant basaloid cells described by Adams et al. [10], with markers including SOX9, NAPSA, ITGB6, and the basal cell transcription factor (TF) TP63. Furthermore, these cells are the primary source of integrin αvβ6 and exhibit high expression of MMP7 and CDKN2A, which are associated with ECM remodeling and epithelial cell senescence, respectively, during the process of pulmonary fibrosis [27, 28]. Notably, this cellular subtype is identified in lung biopsies from asymptomatic individuals with a family history of IPF who show only minimal reticular changes on chest CT, suggesting its early involvement in disease development [12]. Yang et al. [29] also identified a cluster of cells called IPF‐related AT2 (IR_AT2) cells, which are at an intermediate stage of AT2 differentiation, suggesting that they may arise from the activation of resident normal AT2 cells. Their differentiation is accompanied by increased expression of MYC and CDKN2A, indicating their association with cellular senescence. IR_AT2 cells can be further divided into two clusters. IR_AT2 Cluster 1 is linked to HIF1 and IL17 signaling pathways, as well as cellular senescence, emphasizing their role in promoting fibrotic processes. IR_AT2 Cluster 2 is enriched in genes involved in the p53 and Hedgehog signaling pathways. Notably, signaling pathways such as the IL17 and Hedgehog pathways are known to promote fibrosis [30, 31], suggesting that IR_AT2 cells may impact critical fibrotic pathways in the microenvironment. Furthermore, the levels of soluble S100A2 and MMP7 are elevated in IR_AT2 cells, indicating that this AT2 cell subpopulation may play a role in establishing a key profibrotic microenvironment conducive to the differentiation of fibroblasts and myofibroblasts [32, 33]. Additionally, communication between IR_AT2 cells and other subpopulations is characterized by the upregulation of proinflammatory signaling pathways, including macrophage migration inhibitory factor (MIF), growth differentiation factor (GDF), and gamma‐activated sequence (GAS), underscoring the importance of these two subpopulations in IPF pathogenesis.

**TABLE 1:** Epithelial cell subtypes associated with IPF.

Subcluster name	Accumulating locations	Abnormal molecular signaling or biological effects	References
KRT5^−^/KRT17^+^ epithelial cell	Lesions showing high collagen deposition	Fibrosis‐related genes (COL1A1, FN1, CDH2), integrin αvβ6, MMP7, and CDKN2A	[12]
IR_AT2	IPF lungs	MYC, CDKN2A, S100A2, MMP7; HIF1, IL17, p53, Hedgehog, MIF, GDF, and GAS signaling pathways	[29]
GPR87^high^ basal cell	Bronchiectasis and emphysema in IPF tissue	GPR87; PI3K, mTOR, and TNF/NFκB pathways	[34, 35]
Aberrant basaloid cell	Epithelial layer overlying myofibroblast foci in IPF	MMP7, αvβ6 integrin, EPHB2; EMT and cellular senescence	[10]
Cluster B basal cell	Lower lobes of IPF lungs	Convert normal lung fibroblasts into pathogenic myofibroblasts	[36]
ECM basal cell	IPF lungs	ECM components; TGF‐β and SMAD3 signaling pathways	[37]
KRT5^+^/KRT17^+^ basaloid cell	Bronchiectasis and honeycomb cysts	Suppress collagen expression in IPF fibroblasts	[37]
SCGB3A2^high^ club cell	Lungs	Mucins, cytokines, and ECM genes	[38]
MUC5B^+^ club cell	IPF lungs	Genes related to mucus production and immune cell chemotaxis	[38]

In the pathogenesis of IPF, AT2 cells exhibit not only abnormal differentiation states but also alterations in numerous molecular pathways. The expression of the TF *Epas1*, which is associated with the promotion of hypoxic pulmonary hypertension, is significantly altered during the impaired differentiation of AT2 cells [26]. These findings suggest that AT2 cells may contribute to the pathogenesis of pulmonary fibrosis through oxygen‐related pathways [26, 39]. Another important signaling pathway that is activated in AT2 cells during the pathogenesis of IPF is the TGF‐β signaling pathway. Enomoto et al. [24] discovered that the autocrine positive feedback loop of TGF‐β signaling in AT2 cells plays a critical role in the noninflammatory pulmonary fibrosis associated with IPF. TGF‐β induces senescence in AT2 cells and the secretion of the senescence‐associated secretory phenotype, a process that further activates the transformation of lung fibroblasts into myofibroblasts, thereby promoting fibrosis. Furthermore, in IPF, TGF‐β signaling also induces the epithelial‒mesenchymal transition (EMT) in AT2 cells through the SMAD2/3 and MAPK signaling pathways, promoting fibroblast expansion and collagen deposition [40, 41]. Additionally, research on the activation of the Hippo signaling pathway in AT2 cells during IPF has become increasingly detailed. The Hippo pathway plays a complex role in the pathogenesis of IPF. Its effector TFs, YAP and TAZ, are crucial for the differentiation and regeneration of AT2 cells [42]. However, in IPF, abnormal activation of the Hippo pathway is associated with impaired epithelial repair, excessive fibrosis, and cellular senescence [43]. This dual effect of the Hippo pathway is attributed to the fact that YAP activation promotes AT2 cell regeneration and lung repair, whereas TAZ may exacerbate fibrosis by inhibiting this process or promoting maladaptive repair [42, 43].

In IPF, AT2 cells also exhibit numerous biological functional alterations associated with disease progression. AT2 cell senescence is closely associated with the development of IPF. Knockout (KO) of specific genes, such as *Lonp1*, in AT2 cells induces cellular senescence and a reduction in cell number, accompanied by the activation of senescence markers such as *p53* and *p21*. These alterations significantly exacerbate pulmonary fibrosis in mouse models [15, 44]. The potential mechanism linking AT2 cell senescence to the pathogenesis of IPF is primarily endoplasmic reticulum (ER) stress. Borok et al. [45] analyzed tissue samples from IPF patients and animal models and showed that *Grp78*, a key regulator of ER homeostasis, plays a critical role in the ER stress response of AT2 cells in IPF. Moreover, inhibiting ER stress in AT2 cells significantly reversed IPF‐related phenotypes. Additionally, dysfunctional autophagy in AT2 cells may be associated with the pathogenesis of IPF. During BLM‐induced injury, autophagy in AT2 cells promotes alveolar repair by downregulating lipid metabolism and upregulating glucose metabolism. The absence of autophagy‐related protein 5 in AT2 cells exacerbates BLM‐induced lung injury [46].

In summary, the dysregulation of AT2 cells, including impaired differentiation, cellular senescence, and the activation of key signaling pathways such as the TGF‐β, Hippo, and ER stress pathways, contributes to the progression of IPF. The landscape of AT2 cells in the pathogenesis of IPF has been extensively analyzed and characterized. Accelerating the development and validation of therapies targeting AT2 cells and related molecular targets based on existing research is crucial, which will help bridge the gap between scientific research and clinical applications.

#### 2.1.3 Functions and Heterogeneity of Basal Cells in IPF

Basal cells are key stem cells that play crucial roles in maintaining airway homeostasis and repairing airway epithelial damage. These cells, which are located primarily in the basal layer of the airway epithelium, are multipotent and can differentiate into various types of airway cells, including ciliated, secretory, and club cells, depending on the need for repair or regeneration after injury [47, 48].

Recent studies have revealed that basal cells exhibit altered expression patterns and biological behaviors in IPF. ScRNA‐seq of bronchial brush biopsies revealed the dedifferentiation reprogramming of IPF airway basal cells (ABCs) characterized by high expression of KRT17 and low expression of PTEN [49]. This transition enables these cells to form more bronchospheres in three‐dimensional culture models, promoting the proliferation of fibroblasts and the deposition of ECM. Additionally, when implanted into the airways of murine models, these cells induce severe fibrosis, alveolar compartment remodeling, and cystic structure formation [49]. Moreover, several highly expressed molecules in IPF basal cells are associated with disease progression. The expression of GPR87, a G protein‐coupled receptor associated with abnormal cell cycle regulation [50, 51], is increased in IPF basal cells [34, 35]. These GPR87^hi^ basal cells are predominantly located in regions of bronchiectasis and emphysema in IPF tissue sections. Notably, the overexpression of GPR87 impairs the differentiation of KRT5^+^ basal cells into airway cells, which can be induced by TGF‐β treatment in vitro [35]. Khoury et al. [34] further confirmed that increased GPR87 expression promotes fibrosis by activating downstream pathways, including the PI3K, mTOR, and TNF/NFκB pathways. Furthermore, the expression of SNCA, an oxidative stress‐related gene overexpressed in the basal cells of IPF lungs, is strongly correlated with the pathogenesis and poor prognosis of IPF [52]. However, the specific biological function of this gene in basal cells and its mechanistic role in IPF remain unclear. Takashima et al. [53] further identified the expression of the upstream lncRNA MIR205HG in basal cells during the pathogenesis of IPF. MIR205HG is highly expressed in the basal cells of IPF patients and induces an increase in the number of type 2 innate lymphoid cells (ILC2s) by upregulating interleukin (IL)‐33 expression. Activated ILC2s can directly induce fibroblasts to produce collagen, promoting the development of IPF [54]. These results suggest that basal cells contribute to the progression of fibrosis through the regulation of IL‐33 expression by the lncRNA MIR205HG.

In recent years, advances in single‐cell technology have greatly enhanced the cellular resolution of IPF pathogenesis, leading to the identification of various aberrant basal cell subpopulations and variants associated with the development of IPF (Table 1). Adams et al. [10] identified a cluster of aberrant basaloid cells expressing basal cell markers, including TP63, KRT17, LAMB3, and LAMC2, but lacking other well‐known basal cell markers, such as KRT5 and KRT15. These cells are located in the epithelial layer overlaying myofibroblast foci in IPF tissues and exhibit markers of the EMT and cellular senescence. Several IPF‐associated molecules, including MMP7 [55], αvβ6 integrin [56], and EPHB2 [57], are highly enriched in aberrant basaloid cells, suggesting their crucial role in the pathogenesis of IPF. Another subcluster of basal cells that contributes to fibrosis in IPF is called Cluster B basal cells [36]. Characterized by high expression of CXCL17, CEACAM6, IL1RN, and CLDN4, the number of Cluster B cells was significantly increased in IPF patients, and these cells were more abundant in the lower lobes of IPF lungs, which exhibit more severe pathological changes. In vitro and murine model studies have demonstrated that Cluster B cells can convert normal lung fibroblasts into pathogenic myofibroblasts and promote fibroblast recruitment. Certain basal cell subpopulations are present in both healthy individuals and IPF patients but exhibit abnormal expansion and distinct biological processes in IPF patients. For example, using single‐cell sequencing data, Jin et al. [37] identified that ECM basal cells can produce ECM components such as collagen. The proportion of these cells is significantly increased in IPF patients, accompanied by the activation of the TGF‐β and SMAD3 signaling pathways. These findings indicate their potential roles in ECM remodeling and the progression of IPF.

In contrast, basal cell subpopulations may play a negative regulatory role in the progression of IPF. KRT5^+^/KRT17^+^ basaloid cells are primarily found in the pathological remodeling areas of bronchiectasis and honeycomb cysts [58]. The conditioned medium from these basaloid cells suppressed collagen expression in IPF fibroblasts while increasing the secretion of the antifibrotic factor HGF and upregulating MMP‐1 expression. In a murine model of BLM‐induced pulmonary fibrosis, the intratracheal instillation of KRT5^+^/KRT17^+^ basaloid cell‐conditioned medium significantly reduced collagen levels, α‐SMA expression, and lung structural damage, suggesting that these cells regulate abnormal fibroblast activation.

Overall, although the primary lesions in IPF predominantly affect the distal airway epithelium, basal cells in the proximal conducting airways also undergo extensive transcriptomic changes, contributing to the emergence of numerous aberrant subpopulations that have complex and dual effects on disease progression. While changes in the expression patterns and upstream regulatory mechanisms of basal cells in IPF have been elucidated to some extent, further exploration of potential therapeutic targets and validation of their efficacy is warranted. On the other hand, the specific molecular mechanisms of aberrant basal cell subpopulations remain to be validated through in vivo and in vitro experiments, which provides us with access to a comprehensive landscape of various basal cell subclusters that play roles in IPF.

#### 2.1.4 Functions and Heterogeneity of Club Cells in IPF

Club cells (also known as Clara cells) are nonciliated epithelial cells located in the terminal bronchioles of the human airway that play multiple essential roles, including secretion, immune modulation, and tissue repair [59]. Although club cells have received relatively little attention in studies of IPF pathogenesis, recent research has confirmed their contribution to the development of IPF.

The depletion of club cells with naphthalene in BLM‐induced murine models downregulated the expression of genes related to inflammation and chemokine activity in the bronchiolar epithelium [60]. Moreover, club cells exhibit pleomorphism and an abnormal arrangement in IPF lungs [61]. In contrast with those in control samples, the abnormally arranged club cells in IPF lungs often colocalize with α‐smooth muscle actin (α‐SMA), a molecule closely associated with the pathogenesis of IPF [62]. These findings suggest that club cells may contribute to the development of IPF to some extent. The molecular mechanisms through which club cells mediate pulmonary fibrosis in IPF are linked primarily to programmed cell death 5 (PDCD5) [63]. As a molecule whose expression is upregulated in the club cells of IPF patients, PDCD5 can form a complex with β‐catenin and Smad3, promoting the TGF‐β‐induced transcriptional activation of matrix genes and thereby increasing fibroblast proliferation and collagen synthesis. The deletion of *Pdcd5* in club cells significantly alleviates pulmonary fibrosis in a murine model.

Zuo et al. [38] further identified two subpopulations of SCGB1A1^+^ club cells in individuals with IPF, each characterized by distinct expression profiles (Table 1). One subpopulation highly expresses MUC5B, a significant genetic risk factor strongly associated with IPF, whereas the other exhibits heterogeneous expression of SCGB3A2, a marker of club cells. The proportion of MUC5B^+^ club cells is significantly increased in IPF patients, and these cells exhibit high expression of genes related to mucus production and immune cell chemotaxis. In contrast, the proportion of SCGB3A2^high^ club cells remains unchanged in IPF patients, but their molecular phenotype is notably altered, with the significant upregulation of mucins, cytokines, and ECM genes. These findings provide new insights into the biological functions of club cells in the pathogenesis of IPF. However, similar to scRNA‐seq studies of basal cells, the mechanisms through which these subpopulations play roles in the pathogenesis of IPF remain unclear. The precise role of club cells in the development of IPF is still unclear and requires further investigation to elucidate their dynamic changes and associated molecular biological mechanisms, which could provide new cellular targets for IPF treatment.

### 2.2 The Executors: Fibroblast and Myofibroblast Heterogeneity

Fibroblasts and myofibroblasts, as direct executors of fibrosis in the pathogenesis of IPF, are driven by signals such as epithelial injury and repair dysfunction to execute fibrotic programs [64, 65]. During this process, the expression profiles and biological functions of these cells undergo significant alterations compared with their normal state. Recent advancements in scRNA‐seq studies have elucidated the heterogeneity of fibroblast populations and their phenotypic alterations during disease pathogenesis. Understanding the different fibroblast subtypes and their molecular characteristics is crucial for identifying potential therapeutic targets and improving our understanding of IPF.

#### 2.2.1 Altered Expression Patterns of Fibroblasts in IPF

**FIGURE 3:**
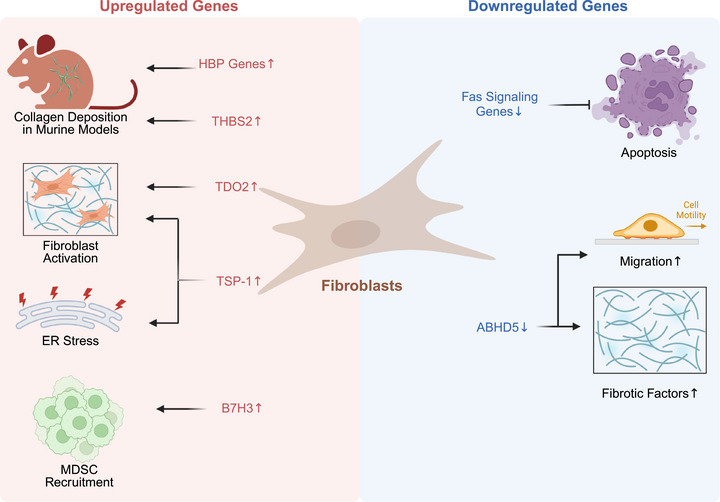
Critical DEGs in IPF fibroblasts identified by single‐cell sequencing. Murine models have validated that upregulated HBP genes and THBS2 promote collagen deposition in IPF. Overexpression of TDO2 and TSP‐1 in IPF correlates with fibroblast activation, with TSP‐1 additionally mediating ER stress responses. Elevating B7H3 expression facilitates MDSCs recruitment, while downregulation of Fas signaling components impairs fibroblast apoptosis, leading to pathological persistence of activated fibroblasts. Fibroblasts with low ABHD5 expression exhibit enhanced migration and secretion of fibrotic factors. *Abbreviations*: DEGs, differentially expressed genes; HBP, hexosamine biosynthetic pathway; ER stress, endoplasmic reticulum stress; MDSCs, myeloid‐derived suppressor cell.

Recent scRNA‐seq studies have provided deeper insights into the changes in gene expression in fibroblasts during IPF progression (Figure 3). Several genes associated with disease development are upregulated in IPF fibroblasts, including B7H3, TDO2, TSP‐1, THBS2, CFH, FHL2, and genes in the hexosamine biosynthetic pathway (HBP) [66, 67, 68, 69, 70, 71]. Secreted B7H3 mediates the recruitment of bone marrow‐derived myeloid‐derived suppressor cells (MDSCs) to the lungs, contributing to fibrosis and inflammatory responses [66]. TDO2 is involved in TGF‐β‐induced fibroblast activation and potentially exacerbates senescence and persistent pulmonary fibrosis through the p53 pathway [67]. TSP‐1 promotes ER stress and fibroblast activation through CD47‐dependent ROS production [69]. THBS2 is a hub gene of fibroblasts in IPF. The inhibition of THBS2 results in the downregulation of fibrosis‐related proteins, such as α‐SMA, fibronectin and collagen I, in BLM‐induced murine models [72]. CFH and FHL2 are upregulated in fibroblasts that express ACTA2 and COL1A1, which are considered primary markers of fibrotic pathology in IPF. Thus, CFH and FHL2, which are precisely localized in IPF fibroblasts, can serve as more accurate indicators of fibroblast foci in individuals with IPF [70]. HBP is critical for the production of O‐GlcNAc. In human lung fibroblasts, the O‐GlcNAc modification regulates the phosphorylation of Smad3 by TGF‐β, thereby increasing the phosphorylation of Smad3. The expression of HBP genes, including OGT, MGEA5, and GFAT1, is significantly upregulated in IPF fibroblasts and myofibroblasts, suggesting that fibroblasts may influence the progression of IPF by modulating the expression of HBP‐related genes [73, 74].

The loss of certain genes, such as those related to Fas signaling and ABHD5, leads to a persistent profibrotic phenotype in fibroblasts, accelerating fibrosis progression. ABHD5 expression is significantly reduced in IPF lung tissue, particularly in fibroblasts. Fibroblasts with low ABHD5 expression exhibit increased migration and secretion of fibrotic factors, accelerating the progression of fibrosis [75]. Additionally, in BLM‐induced murine models, the loss of Fas signaling in fibroblasts affects their apoptosis, resulting in a persistent profibrotic phenotype. Fas signaling is essential for the resolution of fibrosis under physiological conditions, and the loss of Fas‐induced apoptosis may represent a critical checkpoint in the transition from physiological to pathological fibrosis in IPF [76].

In addition to the changes in the expression of the molecules mentioned above, another prominent feature of fibroblasts in patients with IPF is the presence of many transitional fibroblasts in an intermediate stage of differentiation. These transitional cells are typically short lived and present in low proportions, making the assessment of their presence through conventional experimental methods in both in vivo and in vitro samples challenging. However, the high‐resolution capability of single‐cell sequencing has provided crucial support for identifying these important new cell subpopulations (Table 2). Sfrp1^+^ fibroblasts are characterized by high expression of *Sfrp1* and *Col28a1* and represent a noninvasive transitional fibroblast state in the pathogenesis of IPF. These noninvasive fibroblasts were present mainly in the early, relatively less affected regions of the diseased lung. Elevated *Sfrp1* expression in these cells partially inhibits TGF‐β1‐induced fibroblast invasion through the modulation of the RHOA pathway, thereby reducing the invasiveness of fibroblasts during fibrosis progression [77]. The emergence of this subtype reflects the negative regulatory response to the lesion during the early stages of the disease. Another transitional fibroblast subtype, CTHRC1^+^ fibroblasts, which are typically found in fibrotic lungs, is characterized by increased expression of type I collagen (COL1A1) and other ECM‐related genes, such as TNC, POSTN, and COL3A1 [11]. Immunostaining and in situ hybridization revealed that these CTHRC1^+^ fibroblasts are present in fibrotic lesions known as collagen‐producing hotspots during the pathogenesis of IPF [78], suggesting that CTHRC1^+^ fibroblasts may contribute to pathological fibrosis. In murine models of pulmonary fibrosis, CTHRC1^+^ fibroblasts exhibit a heightened migratory ability and high potential for engraftment when transferred to injured lungs [11], reflecting the high degree of tumor invasion associated with CTHRC1 expression [79, 80]. The invasiveness of these fibroblasts promotes pulmonary fibrosis, further supporting the critical role of CTHRC1^+^ fibroblasts in IPF pathogenesis. Additionally, in murine models of pulmonary fibrosis, a transitional state of lipofibroblasts, representing a spectrum between normal lipofibroblasts and myofibroblasts, was identified [81]. Lipofibroblasts in both normal and injured lungs share common transcriptomic characteristics, including typical markers such as *Plin2*, *Wnt2a*, and *Col13a1* [82, 83], as well as genes related to both lipogenic pathways and fibroblast function. In injured lipofibroblasts, notable downregulation of genes linked to adipogenesis and antiaging, such as *Ppp1r15a*, was detected. Conversely, genes associated with fibrosis and senescence, including Serpine2, were upregulated [81, 84]. Fibroblasts with senescent phenotypes contribute to the progression of fibrosis in IPF [85, 86], suggesting that the damage to lipofibroblasts plays an important role in the development of pulmonary fibrosis.

**TABLE 2:** Novel Fibroblast subtypes associated with IPF.

Subcluster name	Abnormal molecular signaling	Associated biological effects	References
F3^+^ fibroblasts	IL‐17 and TNF signaling pathways	/	[87]
ROBO2^+^ fibroblasts	PI3K‐AKT, focal adhesion signaling pathways	EMT and apoptosis	[87]
ECM fibroblasts	ECM genes	EMT	[37]
Inflammatory fibroblasts	FOS, ATF3, and FOSB	/	[37]
TGF‐β signaling‐related fibroblasts	CXCL14, Hedgehog pathway, and TGF‐β signaling pathway	/	[37]
IPF‐fibroblasts	LTBP1, DPT, INHBA, CTHRC1, and TGF‐β response pathways	Negative immune regulation and ECM deposition	[88]
Invasive fibroblasts	HAS1, HAS2, FBN1, and CXCL14	Pathological remodeling in early stage	[10]
Sfrp1^+^ fibroblasts	SFRP1 and COL28A1	Inhibiting TGF‐β1‐induced fibroblast invasion	[77]
CTHRC1^+^ fibroblasts	COL1A1, TNC, POSTN, and COL3A1	Increased invasiveness	[11, 89]
Transitional lipofibroblasts	Plin2, Wnt2a, Col13a1, and Serpine2	Senescent phenotype	[81]
Meflin‐positive fibroblasts	Meflin	Negatively regulating TGF‐β‐induced cellular senescence and fibrosis	[90]

Although single‐cell transcriptomics has provided profound insights into the heterogeneity and transitional states of fibroblasts, these studies are largely limited to small‐scale animal models or in vitro experiments, with a lack of validation using large clinical samples. Furthermore, while several key genes associated with fibrosis progression, such as TDO2, THBS2, and CTHRC1, have been identified in fibroblasts, their precise mechanisms of action and interactions remain unclear. Additionally, most current studies have not elucidated the dynamic changes in these molecular markers at different stages of fibrosis. Therefore, future research should focus on establishing a more precise molecular marker profile to facilitate early diagnosis and monitoring of IPF progression. Moreover, the process of the fibroblast‐to‐myofibroblast transformation and the impact of cellular senescence play crucial roles in the progression of IPF, yet effective interventions targeting these processes remain unclear. Currently, therapeutic approaches targeting fibroblast function are lacking in clinical practice, particularly interventions for the early stages of fibrosis. Consequently, future studies should prioritize the development of early diagnostic tools and precise therapeutic targets, especially those related to senescence, cell migration, and ECM remodeling.

#### 2.2.2 Impact of the ECM on Fibroblast Activation

The critical role of the ECM in fibrosis has long been recognized. In IPF, increased stiffness and synthesis of the ECM contribute to impaired pulmonary function, whereas the mechanical tension attributed to the ECM delivers distinct signals that promote fibroblast activation [91].

The positive feedback loop of the ECM is a key feature in the progression of fibrosis in individuals with IPF. Previous studies have shown that decellularized IPF ECM can induce normal lung fibroblasts to differentiate into activated myofibroblasts, leading to the activation of the translation of ECM proteins (including collagen and other ECM components), which in turn promotes the production of additional ECM and establishes a cycle that further amplifies the fibrotic process. The formation of this positive feedback loop is driven by the downregulation of miR‐29 [92, 93]. miR‐29 is a well‐known negative regulator of ECM genes, and its downregulation leads to increased translation of ECM genes, resulting in the formation of more fibrotic ECM [94]. The overexpression of miR‐29c in the IPF ECM can partially restore pathological gene expression, suggesting that targeting miR‐29 may be a potential therapeutic strategy [93]. Notably, Parker et al. [93] found that compared with cell‐derived signals, ECM‐derived signals play a more crucial role in regulating gene expression, with most changes occurring at the translational level rather than at the transcriptional level. Additionally, increased substrate stiffness activates YAP, which upregulates ECM deposition and stiffens the ECM, thereby promoting the formation of an ECM positive feedback loop [95].

Recent studies have shown that GDF15, one of the most significantly upregulated proteins in IPF lung‐derived ECM, mediates pulmonary fibrosis through fibroblast activation and differentiation [96]. In vivo, GDF15 neutralization in a BLM‐induced lung fibrosis model significantly alleviated lung fibrosis. In vitro, recombinant GDF15 increased the expression of α‐SMA in normal human lung fibroblasts by activating the activin receptor‐like kinase 5 receptor. These findings suggest a potential therapeutic effect of targeting GDF15.

Recent studies on the mechanism of the ECM in the pathogenesis of IPF have provided new insights into its therapeutic approaches. As a crucial intermediate process in the development of IPF, the specific mechanisms of the ECM positive feedback loop warrant further exploration and elucidation. Targeting relevant therapeutic targets to disrupt the ECM positive feedback loop represents a novel strategy for the treatment of IPF.

#### 2.2.3 Function and Heterogeneity of Myofibroblasts in IPF

Myofibroblasts, the most studied subtype of fibroblasts, play a central role in the pathogenesis of IPF [97]. Traditionally, myofibroblasts are characterized by the expression of ACTA2. Single‐cell sequencing has provided a more comprehensive transcriptomic profile of myofibroblasts rather than a single marker [10]. Myofibroblasts in both healthy control and IPF samples expressed common markers such as MYLK, NEBL, MYO10, MYO1D, RYR2, and ITGA8. In IPF, myofibroblasts display increased expression of collagen fibers and ACTA2. Importantly, these myofibroblasts are not a discrete cell type but rather one extreme pole of a continuum connected to ACTA2‐negative stromal cells in healthy lungs [10]. Additionally, peripheral and central lung tissues from IPF patients exhibit distinct histopathological and transcriptomic characteristics. The upregulated differentially expressed genes (DEGs) in central lung tissue are enriched in myofibroblasts, highlighting their central role in the pathogenesis of IPF [98].

Previous studies have precisely defined the transcriptome of IPF myofibroblasts [10, 99]. However, identifying key TFs in upstream regulatory networks based solely on transcriptomics remains imprecise. A study employed single‐nucleus ATAC sequencing (snATAC‐seq) to analyze the chromatin accessibility of myofibroblasts in IPF patients as a method to address this issue [100] and revealed significant enrichment of TWIST1 and other E‐box TF motifs in the open chromatin regions of IPF myofibroblasts. The overexpression of Twist1 led to increased collagen synthesis in IPF myofibroblasts and the upregulation of genes associated with chromatin accessibility in a BLM‐induced murine model.

In summary, myofibroblasts play a pivotal role in IPF pathogenesis, with distinct markers and transcriptomic signatures distinguishing them from other stromal cells. Advances in single‐cell and chromatin accessibility profiling have provided deeper insights into their regulatory networks, identifying key TFs such as TWIST1 that modulate ECM production and fibroblast activation. These findings highlight the potential for targeting transcriptional regulators in therapeutic strategies aimed at controlling myofibroblast‐driven fibrosis in IPF.

#### 2.2.4 Function and Heterogeneity of Novel Fibroblast Subtypes in IPF

High‐resolution single‐cell sequencing has revealed numerous fibroblast subtypes that may play a role in promoting IPF, along with their associated molecular characteristics (Table 2). Zhao et al. [87] identified two subclusters of fibroblasts: F3^+^ fibroblasts and ROBO2^+^ fibroblasts. The former subcluster, characterized by high expression of F3, is closely associated with the IL‐17 and TNF signaling pathways, which are known to regulate fibroblast activity and disease progression in IPF [101, 102]. The latter fibroblast subcluster is characterized by high expression of ROBO2 and is typically found in IPF [87]. Additionally, ROBO2^+^ fibroblasts are linked to the PI3K–AKT and focal adhesion signaling pathways, both of which play crucial roles in the EMT and apoptosis during IPF progression [103]. Jin et al. [37] identified another three fibroblast subclusters associated with IPF progression. ECM fibroblasts, which are characterized by the expression of markers such as POSTN, CTHRC1, and LRRC17, are located primarily around the airways of IPF patients and are characterized by the overexpression of ECM genes such as COL1A1, COL3A1, and COL1A2 [37]. The highly enriched genes TCF4 and MAFB in ECM fibroblasts were previously shown to be associated with renal fibrosis and the EMT [104, 105], indicating a crucial role for ECM fibroblasts in promoting fibrosis in IPF. The second subset, named inflammatory fibroblasts, expresses high levels of FOS, ATF3, and FOSB [37]. Previous studies have shown that inflammatory fibroblasts are associated with high‐level fibrotic Crohn's disease and lung fibrosis [106, 107], indicating that inflammatory fibroblasts may play important roles in IPF pathogenesis. The third subpopulation identified by Jin et al. [37] is called TGF‐β signaling‐related fibroblasts. This cell population is significantly increased in IPF and strongly activated the TGF‐β signaling pathway [37]. Furthermore, these cells are characterized by high expression of CXCL14, which is a systemic biomarker associated with increased Hedgehog pathway activity in IPF patients [108]. Another fibroblast subcluster called IPF fibroblasts is enriched in hub genes, including LTBP1, DPT, INHBA, and CTHRC1, which are related to ECM deposition and the TGF‐β response pathway [88]. Moreover, IPF fibroblasts are negatively correlated with the activation of pathways involved in the immune response, suggesting their potential immunoregulatory role within the fibrotic lung microenvironment [88]. Additionally, invasive fibroblasts, characterized by the expression of markers such as HAS1, HAS2, FBN1, and CXCL14 [10], are thought to contribute to pathological remodeling in IPF. These fibroblasts are particularly abundant in subpleural regions, where fibrotic foci first develop during the progression of pulmonary fibrosis [12].

The progression of IPF is characterized by an imbalance between positive and negative regulatory mechanisms. Therefore, fibroblast subtypes that play negative regulatory roles in disease progression are equally important; however, they have received less attention in related studies. Similar to the Sfrp1^+^ fibroblasts identified by Mayr et al. [77], another subset of fibroblasts that play a negative regulatory role in the pathogenesis of IPF has been discovered. These cells, which are predominantly found in the fibroblast lesions of IPF lungs, are characterized by high expression of ISLR, which encodes meflin [90]. Nakahara et al. [90] reported that these meflin‐positive fibroblasts may exert antifibrotic effects. In meflin‐KO BLM‐induced murine models, the absence of meflin in fibroblasts leads to exacerbated fibrosis, indicating the protective role of meflin‐positive fibroblasts in fibrosis resolution. Additionally, in vitro experiments demonstrated that meflin‐positive fibroblasts negatively regulate TGF‐β‐induced cellular senescence and fibrosis. These results highlight the importance of further studies to increase the expression of meflin on fibroblasts and myofibroblasts in active fibrotic foci in IPF lungs.

The existing research has significantly advanced our understanding of the diverse fibroblast subtypes involved in IPF progression, providing insights into their distinct molecular characteristics and functional roles. However, while these studies have provided valuable insights, several critical gaps remain. First, the functional interplay between these fibroblast subtypes, especially their dynamic transitions during disease progression, remains largely unexplored. Understanding how these subpopulations evolve and interact within complex fibrotic environments is crucial for developing more effective therapeutic strategies. Furthermore, although specific fibroblast subtypes, such as those expressing meflin, have been shown to play potential antifibrotic roles, the mechanisms underlying their regulation and interactions with other cellular components in the fibrotic niche are not fully understood. Another major limitation of the current research is the lack of large‐scale clinical validation of these findings. Most studies rely on small animal models, in vitro experiments, or even only in silico results, which may not fully recapitulate the context of the human disease. Therefore, future research should prioritize the integration of large clinical cohorts and longitudinal studies to validate these fibroblast subtypes and their associated biomarkers in human IPF samples. Additionally, while targeting fibroblast activation and differentiation holds promise, the challenge lies in precisely modulating fibroblast activity without compromising essential physiological functions, such as tissue repair. As such, future therapeutic strategies should aim to selectively modulate fibroblast subpopulations to enhance their protective functions while limiting their contribution to fibrosis.

### 2.3 The Modulators: Immune and Inflammatory Cell Networks

Although IPF is no longer considered an inflammation‐ and immune‐driven disease, increasing attention has been given to the roles of various immune cells, particularly monocytes and macrophages, in the pathogenesis of IPF. As the “regulators” within the pathogenic network of IPF, immune and inflammatory cells are not merely proinflammatory actors; rather, by sensing epithelial stress and matrix mechanical cues and by remodeling the extracellular and metabolic milieu, they amplify or restrain fibrosis across spatial and temporal dimensions. Elucidating the underlying mechanisms and specific impacts of these activities will further improve our understanding of the immune cell landscape associated with IPF pathogenesis.

#### 2.3.1 Alterations in Monocytes in IPF

In patients with IPF, monocytes exhibit marked alterations in both quantity and the functional phenotype [109, 110, 111, 112]. Circulating monocyte counts are significantly greater in IPF patients than in healthy controls, and this increase is negatively correlated with pulmonary function and CT fibrosis scores. Therefore, the monocyte count may serve as a potential biomarker for disease progression in IPF patients [109, 112].

**FIGURE 4:**
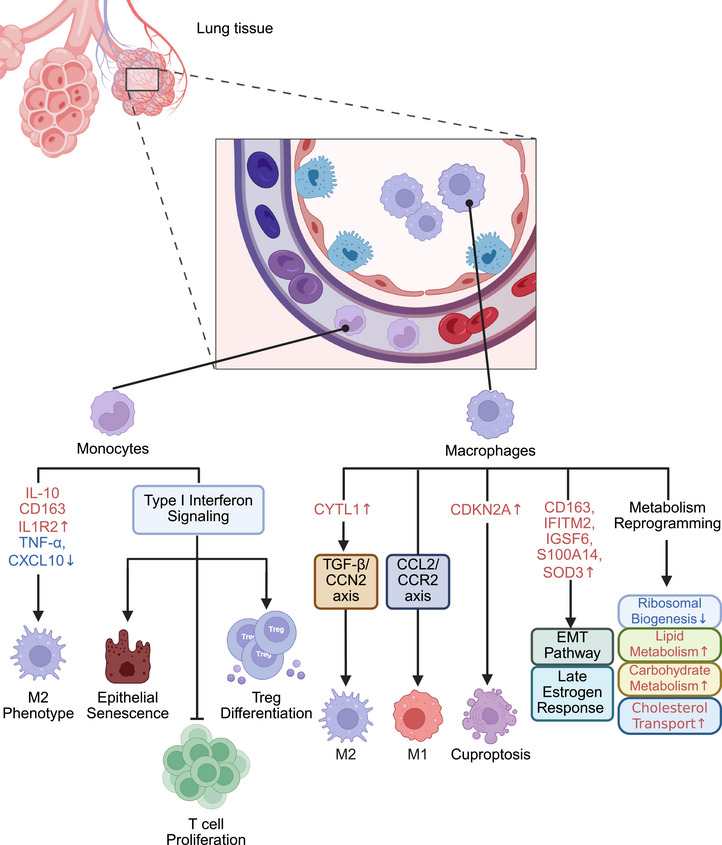
Monocyte–macrophage reprogramming in IPF. In IPF, monocytes exhibit an M2 profibrotic/prorepair phenotype with robust activation of type I interferon signaling. This signaling activation of type I interferon signaling in monocytes significantly suppresses T cell proliferation, promotes Treg differentiation, and induces epithelial cell senescence. Macrophages in IPF undergo metabolic reprogramming, characterized by downregulation of ribosomal biogenesis and upregulation of lipid metabolism, carbohydrate metabolism, and cholesterol transport. Elevated CYTL1 expression in macrophages promotes M2 polarization through the TGF‐β/CCN2 axis, whereas enhanced M1 polarization is driven by the CCL2/CCR2 axis. Additionally, macrophages show upregulation of CD163, S100A14, SOD3, IFITM2, and IGSF6, which are associated with EMT and the estrogen response late signaling pathway, as well as CDKN2A, which is linked to cuproptosis.

With respect to alterations in the functional phenotype, IPF monocytes display prorepair M2‐like characteristics (Figure 4), including increased expression of IL‐10, CD163, and IL1R2 and decreased expression of TNF‐α and CXCL10, suggesting that monocytes in IPF patients may be more involved in aberrant tissue repair processes [109]. Moreover, a prominent feature of monocytes in IPF patients is heightened type I interferon signaling. Fraser et al. [110] found that monocytes from IPF patients exhibit elevated CD64 expression and increased transcription of type I interferon‐related genes, features that correlate with the severity of lung fibrosis. These “immunologically supercharged” monocytes may act as drivers of chronic fibrogenesis in IPF patients [110]. In addition, IPF monocytes display pronounced immunosuppressive properties, which may contribute to the creation of an immunosuppressive microenvironment through the inhibition of T‐cell proliferation and the promotion of regulatory T‐cell differentiation, thereby exacerbating fibrosis progression [111]. Furthermore, Fraser et al. [113] observed an increased presence of a specific myeloid cell cluster with transitional monocyte–macrophage characteristics in IPF lungs. This cluster was characterized by elevated expression of CD64, CD14, and CCL2 and was enriched in type I IFN signaling gene sets. Enhanced downstream innate and adaptive immune responses in IPF patients are associated with chronic inflammation and fibrosis. Additionally, type I IFN can promote epithelial cell senescence [114], a key factor in IPF pathogenesis, by amplifying DNA damage responses and activating the p53 pathway. Karampitsakos et al. [115] further highlighted a unique transcriptional profile in CD14^+^ CD163^−^ HLA‐DR^low^ monocytes, which strongly correlated with the prognosis of IPF patients. Compared with that in patients with stable IPF and controls, the proportion of CD14^+^CD163^−^HLA‐DR^low^ monocytes significantly increased in patients with progressive IPF. A gene signature derived from CD14^+^ CD163^−^ HLA‐DR^low^ circulating monocytes, consisting of 230 genes, was able to predict IPF mortality. Several genes from the gene signature cluster are linked to forced vital capacity (FVC) and have been identified as prognostic biomarkers for IPF in peripheral blood, lung tissue, and bronchoalveolar lavage fluid. These findings underscore the potential role of monocytes in the pathogenesis of IPF.

In the pathogenesis of IPF, monocytes are not directly involved at the site of injury. Therefore, monocytes have been identified as biomarkers for IPF, but research on their specific mechanisms and contributions to IPF is limited. As monocytes are precursors of interstitial macrophages (IMs) in the lung and crucial participants in systemic immune and inflammatory responses, future research should further clarify the role of monocytes in IPF, particularly during the early stages of the disease. Such insights could provide novel avenues for early intervention and monitoring of IPF.

#### 2.3.2 Altered Expression Patterns of Macrophages in IPF

Pulmonary macrophages can be classified into two subsets: alveolar macrophages (AMs) and IMs. AMs are derived primarily from the fetal liver and maintain themselves through self‐renewal under homeostatic conditions, whereas IMs originate from circulating monocytes and play important roles in the lung tissue [116]. The polarization state of pulmonary macrophages determines their functional role in immune responses. In the classical M1 polarization state, macrophages exhibit potent proinflammatory activity that facilitates pathogen clearance, whereas in the M2 polarization state, they display anti‐inflammatory properties that promote tissue repair [117].

After polarizing into different phenotypes, macrophages participate in various processes, such as inflammation and fibrosis, in IPF. Therefore, targeted therapies for IPF should focus on specific targets related to macrophage polarization. In the early stages of IPF pathogenesis, M1 macrophages secrete proinflammatory cytokines and mediate initial lung injury and fibrosis, whereas M2 macrophages promote tissue repair and suppress excessive inflammatory responses through the release of anti‐inflammatory cytokines. However, as the disease progresses, M2 macrophages begin to secrete various fibrogenic mediators, thereby contributing to the progression of fibrosis [118]. Recent studies have shown that in BLM‐induced mice, high expression of CYTL1 regulates macrophage M2 polarization via the TGF‐β/connective tissue growth factor (CCN2) axis, thereby mediating BLM‐induced lung injury and fibrosis. The inhibition of CYTL1 expression significantly suppresses macrophage polarization and improves lung damage [119]. Moreover, Li et al. [120] detected an increased number of M1 macrophages in the pulmonary parenchyma of IPF patients, which may be mediated by the activation of the CCL2/CCR2 axis. Additionally, a specific macrophage subpopulation with a mixed M1/M2 phenotype has been observed in the fibrotic lungs of IPF patients and is characterized by high expression of PLA2G7 [121, 122]. Compared with PLA2G7^low^ macrophages, PLA2G7^high^ macrophages exhibit increased expression of inflammation‐associated genes, such as PLA2G7, IL4I1, STAT1, CXCL9, CCL2, HAMP, and CCR5. These macrophages also show heightened myeloid cell activation and antigen‐processing functions [121, 122]. Notably, M1‐associated markers and M2‐associated genes are both upregulated in PLA2G7^high^ macrophages. This dual activation suggests that PLA2G7^high^ macrophages are functionally plastic. Further experiments revealed that PLA2G7^high^ macrophages contribute to the progression of fibrosis by promoting the fibroblast‐to‐myofibroblast transition (FMT) through the LPC/ATX/LPA/LPA2 axis in macrophages, with PLA2G7 being directly regulated by STAT1 during this process [121].

In addition to being polarized, macrophages in IPF lungs undergo a series of alterations in molecular expression and associated signaling pathways. High expression of five key macrophage marker genes—CD163, IFITM2, IGSF6, S100A14, and SOD3—has been shown to be associated with differences in the survival of IPF patients [123]. The overexpression of these genes activates the EMT pathway, which is strongly involved in the onset of pulmonary fibrosis. Furthermore, the overexpression of CD163, S100A14, and SOD3 also activates late signaling pathways involved in the estrogen response [123]. These compounds have been shown to inhibit fibrosis progression in other tissues, such as liver fibrosis [124], suggesting a potential therapeutic avenue for IPF. Additionally, CDKN2A, an important cuproptosis‐related gene, is highly expressed in IPF macrophages, and its expression is negatively correlated with the survival of IPF patients [27]. CDKN2A is a fibrosis‐associated gene that has been linked to cellular sensitivity to copper‐induced cell death (cuproptosis), a novel form of cell death caused by excessive intracellular copper accumulation [125, 126, 127].

Metabolic reprogramming in IPF‐associated macrophages involves a dual regulatory axis: the downregulation of ribosomal biogenesis concurrent with the activation of lipid metabolism, including upregulation of genes enriched in cholesterol homeostasis and lipid transport mechanisms. Single‐cell analyses further revealed that this lipid‐centric metabolic signature is predominantly enriched in AMs from IPF patients, indicating that AM‐driven lipid dysregulation is a potential modulator of fibrogenesis. Additionally, DEGs associated with carbohydrate metabolism, including processes such as glycolysis, pyruvate metabolism, and the pentose phosphate pathway, are generally upregulated in monocyte‐derived macrophages (MO‐MACs) in IPF patients [128]. This metabolic reprogramming facilitates rapid energy generation by inflammatory immune cells, with hypoxia‐induced glycolysis playing a crucial role in the survival of MO‐MACs and the activation of pulmonary fibrosis [129, 130]. The expression of the gene encoding enolase 1, a key enzyme involved in the metabolic reprogramming of lung fibrosis [131, 132], is upregulated in both AMs and MO‐MACs from fibrotic lung tissue [128]. Moreover, genes related to cholesterol transport, such as ATP‐binding cassette subfamily A member 1 (ABCA1), are upregulated in AMs [128]. ABCA1 is a key transporter involved in cholesterol transport and has been shown to prevent lung inflammation and fibrosis by promoting surfactant production [133]. Certain specialized macrophage subpopulations associated with metabolic reprogramming have also been identified in individuals with IPF. A distinct macrophage subset exclusively identified in IPF lung tissues, termed ATP5‐MΦ, shows high expression of the ATP5 gene family [134]. An enrichment analysis indicated that ATP5‐MΦs are highly involved in biological processes, including proton transmembrane transport, ATPase complex activity, and oxidative phosphorylation processes. This metabolic reprogramming of macrophages may be related to their specific function in regulating immune responses. Another macrophage subpopulation, P2RX7^+^ macrophages, interacts with ATP to induce lung injury, and neutralizing ATP or blocking P2RX7 can alleviate lung fibrosis [135, 136]. Marked by high P2RX7 expression, P2RX7^+^ macrophages are closely associated with ceramide metabolism and the establishment of an immunosuppressive environment. Additionally, P2RX7^+^ macrophages are related to PDGF‐, FGF‐, and VEGF‐associated pathways during fibrosis [113]. These results further confirm the role of P2RX7^+^ macrophages in the pathogenesis of IPF.

In conclusion, the complex interplay of macrophages in IPF, characterized by distinct metabolic reprogramming and changes in biological functions, highlights their critical role in driving the pathogenesis of the disease. These immune cells not only contribute to fibrosis through mechanisms such as macrophage polarization and inflammatory signaling but also modulate interactions with other cells in the lung microenvironment. Understanding the molecular pathways that govern these cellular processes may provide valuable insights into new therapeutic strategies aimed at modulating macrophage function and improving the outcomes of patients with IPF.

#### 2.3.3 Function and Heterogeneity of SPP1^hi^ Macrophages in IPF

Among the macrophage subpopulations identified through scRNA‐seq, SPP1^hi^ macrophages have garnered significant attention as central players in IPF pathogenesis because of their prominent role in fibrosis progression. Initially identified by Morse et al. [137], SPP1^hi^ macrophages exhibit low proliferation rates in normal lungs but markedly expand in IPF‐affected tissues. A trajectory analysis revealed that these cells are at the end of the developmental trajectory and originate from CD14^+^ monocytes rather than tissue‐resident AMs [138, 139]. These macrophages are characterized by elevated expression of both *SPP1* and *MERTK*, with a particularly pronounced upregulation observed in the lower lobes of fibrotic lungs [137]. The increased expression of both *SPP1* and *MERTK* is believed to be key for understanding their involvement in tissue repair and fibrosis processes. MERTK serves as a receptor for Gas6 or Protein S, both of which bind to phosphatidylserine on apoptotic cells [140, 141] and are crucial for the recognition of apoptotic cells by macrophages [142, 143]. Alveolar cell apoptosis is a hallmark of IPF [144], and blocking Gas6 has been shown to inhibit the expression of markers of fibroblast activation [145]. Colocalization and causal models support that these highly proliferative SPP1^hi^ macrophages promote myofibroblast activation, thereby exacerbating lung fibrosis [137].

Increased expression of SPP1 is primarily observed in M2 macrophages in IPF tissues [146]. Interestingly, the specific expression of SPP1 can inhibit the proliferation and migration of M2 macrophages and promote their apoptosis. These unexpected results may be due to an in vivo negative feedback mechanism or differences between in vitro and in vivo environments, including differences in receptor expression, the cellular activation status, and other signaling molecules [146]. Furthermore, the overexpression of SPP1 in macrophages may promote the progression of IPF to lung cancer. IPF patients with high SPP1 expression exhibit an immunosuppressive TME and high oncogene expression. In vitro experiments showed that coculturing SPP1^hi^ macrophages with MRC‐5 cells induced the transformation of fibroblasts into cancer‐associated fibroblasts. Macrophage‐derived SPP1 promoted the EMT in alveolar epithelial cells by stimulating the upregulation of N‐cadherin and vimentin expression in MLE‐12 cells. Single‐cell sequencing data indicate that communication between SPP1^hi^ macrophages and inflammation‐associated cancer‐associated fibroblasts drives the tumorigenic process in IPF [138].

Furthermore, scATAC‐seq has revealed the upstream regulatory mechanisms associated with SPP1^hi^ macrophages [139]. The expression and chromatin accessibility of APOE are increased in SPP1^hi^ macrophages. APOE is involved in lipid metabolism and a risk factor implicated in various fibrotic diseases [147, 148, 149], and its upregulation in the AMs of IPF patients plays a key role in type I collagen phagocytosis [147, 150].

Additionally, the number of SPP1^hi^ macrophages is closely correlated with the clinical prognosis of IPF patients. IPF patients with an increased frequency of SPP1^hi^ macrophages in their lungs exhibit poorer lung function. In the low FVC subgroup, the proportion of SPP1^hi^ macrophages was greater than that in the high FVC subgroup, further suggesting that SPP1^hi^ macrophages contribute to lung fibrosis.

In conclusion, SPP1^hi^ macrophages constitute a distinct monocyte‐derived subset with multifaceted roles in IPF, contributing to fibrogenesis, immune modulation, and potentially tumorigenesis. Their unique molecular signatures, including elevated SPP1, MERTK, and APOE expression, link them to key pathogenic processes such as apoptotic cell clearance, myofibroblast activation, and the EMT. While these findings position SPP1^hi^ macrophages as promising biomarkers and therapeutic targets, the current knowledge is largely based on static single‐cell profiles and in vitro models, which cannot fully capture their dynamic behaviors and context‐dependent functions in vivo. Future studies should employ longitudinal single‐cell multiomics, spatial transcriptomics, and functional perturbations in relevant animal models to clarify the temporal evolution, plasticity, and causal contributions of SPP1^hi^ macrophages to IPF progression. Moreover, understanding the interplay between SPP1^hi^ macrophages, the fibrotic niche, and tumor‐promoting microenvironments is essential for the development of targeted interventions that mitigate fibrosis without compromising tissue repair or host defense.

#### 2.3.4 Other Novel Macrophage Subclusters in IPF

Similar to epithelial cells and fibroblasts, multiple distinct monocyte subpopulations associated with IPF pathogenesis have been identified by single‐cell sequencing (Table 3). Some subtypes have intermediate expression profiles between those of AMs and IMs. For instance, IPF‐expanded macrophages (IPFeMΦs) represent a transitional state between alveolar and IMs and exhibit a differentiation trajectory distinct from that of AMs [151, 152, 153]. These cells show the progressive upregulation of fibrosis‐related genes, including ECM remodeling‐related genes such as metalloproteinases, secreted mediators, proteases, tissue remodeling modulators, and enzymes. Furthermore, IPFeMΦs exhibit differential expression of lipid metabolism‐related genes, including higher expression of LPL, LIPA, NCEH1, and CD36, which distinguishes them from AMs characterized by PARG, FABP4, and FABP5 expression [151]. Aran et al. [154] also identified a disease‐associated macrophage subtype characterized by a gene expression profile intermediate between that of monocyte‐derived and AMs in a BLM‐induced murine model. This subtype, termed Cx3cr1^+^SiglecF^+^ transitional macrophages, is localized to fibrotic niches enriched with Pdgfra^+^ and Pdgfrb^+^ fibroblasts. During the fibrosis phase, the depletion of these macrophages led to a reduced accumulation of Pdgfra^+^ or Pdgfrb^+^ fibroblasts and a significant decrease in the total lung collagen content. Furthermore, fibroblast migration and proliferation were found to depend on Pdgf‐aa, a growth factor in fibrotic niches that is derived mainly from Cx3cr1^+^SiglecF^+^ macrophages.

**TABLE 3:** Novel macrophage subtypes associated with IPF.

Subcluster name	Abnormal molecular signaling	Associated biological effects	References
IPFeMΦ	ECM remodeling‐related genes and lipid metabolism‐related genes	/	[151, 152, 153]
Cx3cr1^+^SiglecF^+^ macrophage	Pdgf‐aa	Inducing fibroblast migration and proliferation	[154]
Profibrotic AMs	CHI3L1, MARCKS, IL1RN, PLA2G7, MMP9, and SPP1	/	[10, 99]
IPF‐MΦ	PPAR signaling and p53 signaling	Shaping immune microenvironment and driving fibrotic progression	[134]
PLA2G7high macrophages	PLA2G7, IL4I1, STAT1, CXCL9, CCL2, HAMP, and CCR5	Fibroblast‐to‐myofibroblast transition	[121, 122]
ATP5‐MΦ	ATP5 gene family	Regulating immune responses	[134]
P2RX7^+^ macrophages	P2RX7	Ceramide metabolism and establishing immunosuppressive environment	[136]

In addition to the transitional states between AMs and IMs, other novel macrophage subtypes associated with IPF have been reported through in silico research. Reyfman et al. [99] identified a profibrotic AM subpopulation characterized by high expression of CHI3L1, MARCKS, IL1RN, PLA2G7, MMP9, and SPP1, which strongly correlated with pulmonary fibrosis. Using an archetype analysis, Adams et al. [10] further explored the characteristics of these IPF‐specific macrophages, identifying a gradual, continuous shift along the IPF macrophage archetype, with SPP1, cholesterol esterase, lipoprotein lipase, and LIPA expression steadily increasing in earlier trajectories. This analysis revealed a steady increase in the expression of ECM remodeling‐related genes, including SPARC, GPC4, PALLD, CTSK, and MMP9, with macrophages at the endpoint of this trajectory expressing CSF1, suggesting the presence of an autocrine feedback loop that recruits and activates additional macrophages. Zhang et al. [134] further identified an IPF‐associated macrophage subset in human lung tissues, termed IPF‐MΦs. Genes that were highly expressed in IPF‐MΦs were enriched in biological functions such as PPAR signaling and p53 signaling. Nine hub genes of IPF‐MΦs, namely, FAM174B, PMP22, ATF4, DLD, ELOB, CTDP1, SV2B, USP10, and PHACTR1, strongly correlated with the IPF prognosis. These macrophages strongly communicate with other macrophages via various ligands and receptors, including GRNSORT1 and MIF‐(CD74 + CD44). Mechanistically, MIF suppresses the random migration and adhesion of monocytes/macrophages while regulating immune responses via the AKT and NF‐κB pathways [155, 156]. GRNSORT1, a lipid metabolism‐associated factor, facilitates cholesterol accumulation in macrophages [157]. Furthermore, IPF‐MΦs displayed enhanced interactions with myofibroblasts, ciliated cells, AT2 cells, epithelial cells, and fibroblasts, which are potentially driven by the ANXA1–FPR2 and NAMPT (ITGA5 + ITGB1) signaling pathways. The ANXA1–FPR2 axis helps maintain the fibroblast balance, with ANXA1, which is overexpressed in macrophages infiltrating damaged tissues, promoting an anti‐inflammatory macrophage phenotype via FPR2–AMPK signaling and suppressing inflammation and myofibroblast regeneration [158, 159]. Collectively, these findings implicate IPF‐MΦs in shaping the immune microenvironment and driving the progression of fibrosis in IPF.

In summary, recent single‐cell transcriptomic studies have revealed substantial heterogeneity among macrophage populations in individuals with IPF. These macrophage subsets not only contribute to ECM remodeling and fibroblast activation through diverse ligand–receptor networks but also engage in complex metabolic and immunoregulatory processes that may sustain chronic fibrosis. However, most evidence available to date is derived from cross‐sectional or animal model studies, limiting causal inference and the temporal resolution of macrophage state transitions. Moreover, functional validation of key signaling axes—particularly those involving lipid metabolism, autocrine feedback, and fibroblast–macrophage crosstalk—remains incomplete. Future research should integrate longitudinal multiomics profiling with targeted in vivo perturbations to clarify the lineage relationships and therapeutic potential of these macrophage subsets in IPF progression.

#### 2.3.5 T Cells

Recent studies have increasingly focused on the distinct role of T cells in the pathogenesis of IPF. T cells are broadly classified into three major subsets—CD4⁺ T cells, CD8⁺ T cells, and γδ T cells—based on their surface markers and T‐cell receptor composition. CD4⁺ T cells can be further subdivided into Th1, Th2, Th17, and Treg subsets according to their distinct functions and cytokine profiles.

In IPF, the CD4⁺ T‐cell subsets that play pivotal roles are primarily Th17 cells and Tregs. Th17 cells act mainly as profibrotic mediators in IPF pathogenesis by secreting IL‐17A, which activates pulmonary fibroblasts and thereby drives fibrosis progression [160]. Recent studies have demonstrated that macrophage‐inducible C‐type lectin (Mincle) promotes Th17 differentiation, initiating IL17‐mediated inflammation and subsequently inducing acute exacerbations of IPF [161]. Furthermore, multiple previous studies have reported that the antifibrotic mechanisms of drugs such as theophylline, daphnetin, and donepezil in IPF involve the inhibition of Th17 differentiation and IL‐17 production [160, 162, 163]. Collectively, these findings highlight the crucial role of Th17 cells in the pathogenesis of IPF. However, a detailed elucidation of the mechanisms underlying Th17 infiltration and its impact is lacking. Future studies should further investigate the upstream triggers of Th17 differentiation and the specific regulatory effects of Th17 cells on fibroblasts and other target cells in IPF.

Tregs are a subset of T cells with immunosuppressive functions. Kotsianidis et al. [164] first provided evidence of impaired Tregs in IPF patients. Compared with those from healthy controls, Tregs from IPF patients exhibited a markedly diminished suppressive capacity, with impaired inhibition of Th1‐ and Th2‐type cytokine secretion. Moreover, this Treg dysfunction was strongly correlated with the severity of IPF. Recent studies have further explored the changes in Tregs in IPF using single‐cell sequencing technology. Unterman et al. [165] observed increases in both the number and the percentage of Tregs among peripheral blood T cells in both patients with stable and progressive IPF, with a more pronounced increase observed in the progressive phase. Notably, this increase in Tregs is particularly striking given the reduction in total lymphocyte counts during disease progression. Notably, patients with higher Treg counts exhibited distinct survival curves, characterized by significantly higher mortality rates, suggesting that Treg levels may serve as a prognostic indicator in IPF. Furthermore, genes related to the Treg activation pathway, including those involved in the TGFβ/SMAD, mTOR, and IL‐2 pathways, were upregulated, whereas those involved in the IFN‐γ pathway were downregulated in the peripheral Tregs of patients with progressive IPF.

Significant changes in Th17 cells and Tregs occur during the pathogenesis of IPF. However, the underlying mechanisms and specific roles of these changes remain unclear. Future research should focus on elucidating how these cellular alterations occur and contribute to the progression of IPF to identify new cellular targets for IPF therapy.

In the lung tissue of IPF patients, the infiltration of CD8⁺ T cells was shown to be correlated with functional parameters indicating disease severity, suggesting a potential role in its pathogenesis [166]. Recent studies have shown that CD8⁺ T cells in IPF patients exhibit a CD28^null^ phenotype, which is closely associated with disease progression and a poor prognosis [167]. Compared with normal CD8⁺ T cells, CD28^null^ T cells in IPF lungs do not differ significantly in the expression of the immune checkpoint protein CTLA‐4 but show a marked increase in PD‐1 expression. Blockade of either immune checkpoint protein significantly accelerated the progression of pulmonary fibrosis in murine models. Furthermore, scRNA‐seq analysis revealed that CD8⁺ T cells in individuals with IPF undergo metabolic reprogramming and are enriched in signaling pathways associated with fibrosis [168]. The major CD8^+^ T‐cell subgroups involved in IPF are the CD8‐FOSB, CD8‐ZNF683, and CD8‐IFNG clusters. A trajectory analysis of these subgroups indicated that early CD8^+^ T cells, primarily CD8‐FOSB cells, were predominantly distributed in IPF samples, with their amino acid‐related metabolic pathways being more active in IPF patients than in controls. In contrast, CD8^+^ T cells in control samples were located at the terminal stage of the cell state transition pathway [168]. CD8‐FOSB cells exhibited characteristics of naive T cells and were thought to have strong proliferative potential [169]. In addition to the heterogeneity of these specific subgroups, metabolic reprogramming of CD8^+^ T cells is also implicated in the pathogenesis of IPF. Specifically, carbohydrate‐related metabolic pathways in the CD8‐FGFBP2, CD8‐ZNF683, and CD8‐IFNG subgroups were more active in IPF patients, whereas lipid‐related metabolic pathways were more active in the CD8‐ZNF683 subgroup. Metabolites in different CD8^+^ T‐cell clusters exhibited similar metabolic patterns, with the consistent downregulation of glucose‐6‐phosphate, methionine, and 5‐aminoimidazole‐4‐carboxamide ribonucleotide, while glutamine, fumarate, oxaloacetate, pyruvate, and 2‐oxoglutarate were upregulated, all of which are associated with a profibrotic phenotype [168, 170, 171, 172, 173].

These findings suggest that CD8⁺ T cells may play a role in promoting the fibrotic process of IPF; however, the precise mechanisms underlying their involvement remain unclear and warrant further studies to confirm and elucidate this notion.

In IPF, γδ T cells primarily exert antifibrotic effects. In a mouse model of BLM‐induced pulmonary fibrosis, the number of γδ T cells increased dramatically, and these expanded γδ T cells promoted the resolution of fibrosis through the production of CXCL10. The deletion of either γδ T cells or CXCL10 in these models significantly worsened the severity of fibrosis and impaired its resolution [174]. Moreover, γδ T cells can regulate collagen type I synthesis in lung fibroblasts both through direct cell‐to‐cell contact and through the secretion of soluble antifibrotic factors, such as IFN‐γ, highlighting a potential mechanism underlying the antifibrotic effects of γδ T cells in individuals with IPF [175]. However, other studies suggest that under certain conditions, γδ T cells play a proinflammatory role in IPF. γδ T cells continue to produce IL‐17A, particularly in the absence of Th17 cells, exacerbating chronic lung inflammation and fibrosis. Additionally, IL‐17A^+^ γδ T cells are associated with the accumulation of neutrophils, increased numbers of M2 macrophages, and exacerbated fibrosis [176]. These findings suggest that different subsets of γδ T cells may play dual roles in the progression of IPF or that γδ T cells may exert distinct effects at different stages of the disease and within various pathological microenvironments. These complex effects and mechanisms warrant further investigations in larger‐scale studies and clinical samples.

#### 2.3.6 Neutrophil

Although numerous studies have suggested that neutrophils may be involved in the pathogenesis of IPF, the exact nature of this relationship remains unclear [177, 178]. In the microenvironment of the IPF lung tissue, neutrophil infiltration is markedly reduced, and neutrophil‐related hub genes have been identified as promising diagnostic biomarkers for IPF [179]. Recent studies have demonstrated increased formation of neutrophil extracellular traps (NETs) in patients with IPF [180]. NETs, a type of DNA meshwork released by neutrophils, can capture pathogens but may also promote fibrosis by activating factors such as TGF‐β. In addition, neutrophils in IPF patients exhibit increased cellular stiffness and an enlarged cell volume, which may increase their retention within the microvasculature, thereby aggravating lung injury and inflammation [181]. Collectively, these findings suggest that neutrophils may play an important role in IPF pathogenesis. However, the precise impacts of alterations in neutrophils and their functions—whether reparative or pathologically destructive to the fragile lung architecture—remain to be elucidated in future studies.

#### 2.3.7 Mast Cells

Mast cells are typically involved in allergic reactions; however, recent studies have revealed that mast cell accumulation and activation play significant roles in promoting fibrosis and disease progression in IPF. In the lungs of IPF patients, the number of activated mast cells increases near fibroblast foci and type II alveolar cells [182]. Upon activation, these accumulated mast cells degranulate and release various profibrotic factors, such as tryptase and TGF‐β1, which activate fibroblasts and drive the progression of fibrosis [183]. Furthermore, Wygrecka et al. [182] discovered that in IPF, a positive feedback loop is established between mast cell activation and fibroblast activation. Tryptase released by activated mast cells can promote fibroblast activation and ECM production through the protease‐activated receptor‐2 (PAR‐2)/PKC‐α/p44/42 signaling pathway, while fibroblasts can increase mast cell survival and proliferation by releasing stem cell factor (SCF), which acts on the c‐Kit receptor on mast cells [182]. Although the existing evidence suggests that mast cells may be involved in the pathogenesis of IPF and cooperate with fibroblasts to promote fibrosis, further studies of the specific molecular mechanisms of this regulatory loop are necessary to identify suitable therapeutic targets for mast cell‐directed treatments for IPF.

### 2.4 Crosstalk in the IPF Niche: Integrating Cellular Mechanisms

**FIGURE 5:**
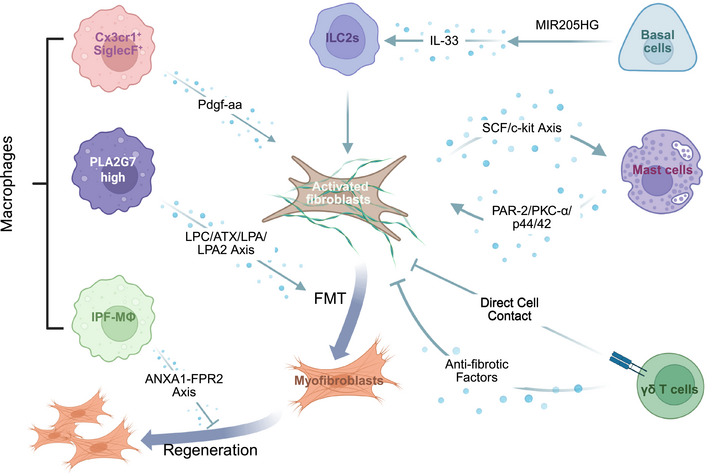
Immune cell–fibroblast crosstalk in IPF. In IPF, Cx3cr1^+^SiglecF^+^ transitional macrophages promote fibroblast activation by producing Pdgf‐aa. PLA2G7‐high macrophages promote the conversion of fibroblasts to myofibroblasts through the LPC/ATX/LPA/LPA2 axis. IPF‐MΦ inhibit myofibroblast regeneration via the ANXA1–FPR2 axis. Basal cells induce elevated IL‐33 levels through high MIR205HG expression, thereby promoting ILC2‐mediated fibroblast activation. Mast cells in IPF form a positive feedback loop with activated fibroblasts. Mast cells activate fibroblasts through the PAR‐2/PKC‐α/p44/42 signaling pathway, while activated fibroblasts secrete SCF to stimulate mast cell c‐kit receptor, promoting their activation. γδ T cells can suppress fibroblast activation either through direct cell contact or by secreting antifibrotic factors. *Abbreviations*: FMT, fibroblast–myofibroblast transition; IPF‐MΦ, IPF‐associated macrophage; ILC2s, type 2 innate lymphoid cells.

With the advancement of omics technologies, such as single‐cell sequencing, our understanding of the abnormal gene expression patterns of different cell types in IPF has deepened. However, these aberrant gene expression profiles are not isolated cellular events, they are part of a dynamic crosstalk network formed by epithelial cells, fibroblasts, and immune cells through various molecular signaling pathways and ligand–receptor interactions within the fibrotic microenvironment (Figure 5). These interactions, driven by signaling transduction, positive feedback loops, and spatial proximity, create a self‐sustaining fibrotic cycle that contributes to the initiation of lung tissue damage, amplification of inflammation, and abnormal deposition of ECM.

#### 2.4.1 Epithelial Cell and Immune Cell Interactions: Injury Initiation and Inflammation Amplification

Pulmonary epithelial cells, particularly AT2 cells, serve as the “initiators” of IPF. Under environmental stressors, such as oxidative stress or mechanical injury, these cells release damage‐associated molecular patterns and reactive oxygen species (ROS), which recruit and activate immune cells, thereby initiating an inflammatory cascade [184, 185]. Following epithelial injury, the secretion of cytokines such as IL‐6, IL‐1β, and TNF‐α is upregulated. These cytokines not only exacerbate the inflammatory response by activating local immune cells, such as macrophages and neutrophils, but also induce the production of chemokines such as CXCL8 and CCL2, which further recruit immune cells into the damaged regions [186, 187]. Macrophages play a pivotal role in this process. Attracted by the chemokines released by damaged epithelial cells, they infiltrate the lung tissue, where they are further activated by signaling molecules like Sonic Hedgehog released by epithelial cells. This activation triggers the JAK2/STAT3 pathway, leading to the secretion of large amounts of TGF‐β, which is involved in the typical fibrotic feedback loop, promoting the progression of fibrosis [188, 189]. On another level of immune signal amplification, thymic stromal lymphopoietin and MMP9 secreted by macrophages can, in turn, promote EMT in epithelial cells, further exacerbating tissue fibrosis [190]. Additionally, other immune cells, such as neutrophils, can activate TGF‐β through the formation of NETs, contributing to the positive feedback loop of fibrosis in IPF [180].

In summary, the interaction between epithelial cells and immune cells forms a positive feedback loop: epithelial injury recruits and activates immune cells, and in turn, the immune cells secrete factors that amplify epithelial apoptosis and senescence, further participating in the fibrotic feedback loop through key signals such as TGF‐β.

#### 2.4.2 Epithelial–Fibroblast Crosstalk: Bridging Injury and Fibrosis Execution

The interactions between epithelial cells and fibroblasts in IPF are considered the core mechanism driving disease progression through a positive feedback loop. Repeated injury to alveolar epithelial cells results in abnormal gene expression patterns and functional impairment. These dysfunctional epithelial cells, in turn, translate the signals of epithelial injury into fibrotic activation through two distinct pathways [191, 192]. On one hand, epithelial cells in IPF undergo EMT and acquire fibroblast‐like characteristics [193]. On the other hand, damaged and senescent epithelial cells secrete a variety of profibrotic factors, which activate adjacent fibroblasts via paracrine signaling and exacerbate epithelial injury and phenotypic changes via autocrine signaling [194, 195]. TGF‐β induces certain alveolar epithelial cells to acquire fibroblast‐like characteristics and directly participate in the formation of fibrotic foci, while stimulating profibrotic factor secretion by aberrant epithelial cells, thereby activating fibroblasts [40, 196]. Activated fibroblasts, in turn, secrete ROS, angiotensin II, and TGF‐β, which exacerbate epithelial damage, creating an “epithelial–fibroblast positive feedback loop” that sustains fibrotic progression [194].

Overall, the interaction between epithelial cells and fibroblasts in IPF forms a positive feedback loop centered around TGF‐β signaling, leading to the exacerbation of fibrosis and progressive loss of lung function.

#### 2.4.3 Immune Cell–Fibroblast Crosstalk: Modulation of Inflammation and Amplification of Fibrosis

In IPF, immune cells act as pivotal “regulators” that orchestrate fibroblast activation through the secretion of profibrotic mediators and metabolic signaling molecules. This immune–fibroblast interaction forms a self‐amplifying inflammatory–fibrotic axis that sustains disease progression. ScRNA‐seq has revealed substantial heterogeneity within this crosstalk, showing that distinct immune cell subsets activate fibroblasts via unique ligand–receptor networks, promoting fibroblast differentiation, proliferation, and ECM remodeling. Among these immune populations, macrophages occupy a central role in mediating fibroblast activation. Cx3cr1⁺SiglecF⁺ transitional macrophages promote fibroblast proliferation and ECM synthesis by secreting Pdgf‐aa [154]. In contrast, PLA2G7‐high macrophages facilitate FMT through the LPC/ATX/LPA/LPA2 signaling axis [121], whereas a subset of IPF‐associated macrophages (IPF‐MΦ) suppress myofibroblast regeneration via the ANXA1–FPR2 pathway [158, 159]. These findings suggest that macrophage subpopulations can exert both pro‐ and antifibrotic effects, reflecting a context‐dependent regulatory balance within the fibrotic niche. Other immune cell subsets also contribute to fibroblast modulation in IPF. Basal cells, through upregulation of the lncRNA MIR205HG, drive increased production of IL‐33, which in turn enhances ILC2 activation and augments fibroblast activation [53]. Mast cells establish a positive feedback loop with fibroblasts: they activate fibroblasts through the PAR‐2/PKC‐α/p44/42 signaling cascade, while activated fibroblasts secrete SCF that binds to the c‐kit receptor on mast cells, reinforcing their activation and perpetuating profibrotic signaling [182]. Conversely, γδ T cells exhibit an antifibrotic role, attenuating fibroblast activation either through direct cell–cell contact or via the secretion of antifibrotic mediators [176].

Collectively, these findings highlight that immune–fibroblast interactions constitute a multifaceted regulatory network driving IPF progression. The balance between profibrotic and antifibrotic immune subsets determines the trajectory of fibroblast activation and matrix remodeling. Dissecting these interactional pathways provides critical insight into disease pathogenesis and may reveal novel therapeutic targets for precise modulation of fibrogenic responses in IPF.

## 3 Spatial Transcriptomics: Mapping the Architecture of Fibrosis

Spatial transcriptomics, a cutting‐edge technology, offers novel perspectives and deeper insights into the mechanisms underlying IPF. While scRNA‐seq can reveal the transcriptional characteristics of cell populations and infer intercellular communication through known ligand‒receptor interactions, these analyses often neglect the spatial proximity of these cells, which is crucial for cellular crosstalk. Given that the pathological features of IPF involve extensive intercellular interactions and structural changes in lung tissues, the lack of spatial information limits a comprehensive understanding of cellular functions and interactions. Spatial transcriptomics overcomes this limitation by capturing and quantitatively analyzing mRNA molecules in situ, thereby providing spatial resolution for cellular populations. This approach preserves the spatial localization of RNA molecules within tissue sections and integrates techniques such as in situ capture, in situ sequencing, or fluorescence in situ hybridization. Without disrupting the native tissue architecture, it enables quantitative and visual analysis of gene expression across spatial dimensions, thereby revealing spatially resolved patterns of transcriptional activity [197]. By integrating scRNA‐seq data with spatial transcriptomics, researchers can now more accurately depict the complex cellular dynamics and spatial organizational patterns within IPF tissue. The application and advancement of this technology have greatly expanded our insight into the mechanisms underlying IPF.

### 3.1 Identification of Pathological Spatial Niches

**FIGURE 6:**
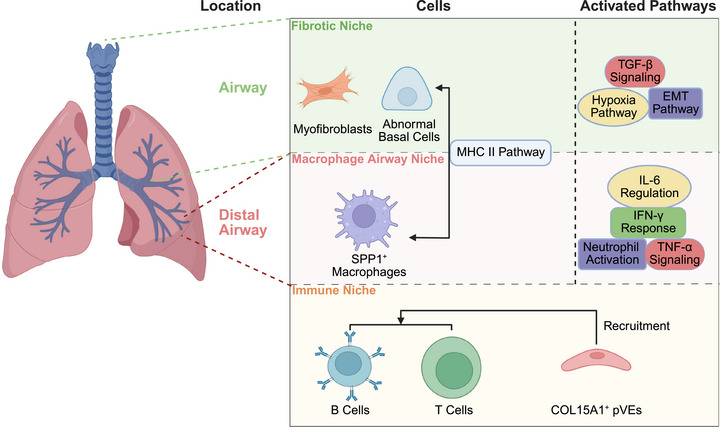
Three IPF‐related niches identified by space transcriptomics. The fibrotic niche, predominantly localized in the airway, is characterized by abnormal basal cells and myofibroblasts, exhibiting robust TGF‐β signaling, hypoxic pathways, and activation of EMT. The airway macrophage niche, located in distal airways, consists primarily of SPP1^+^ macrophages and is marked by strong TNF‐α signaling, IFN‐γ responses, neutrophil activation, and IL‐6 regulation. Notably, abnormal basal cells within the fibrotic niche and SPP1^+^ macrophages in the airway macrophage niche interact closely via MHC II pathway. The immune niche, composed mainly of B and T cells, appears to be recruited by secretory signals from COL15A1^+^ pVEs. pVEs, peribronchial vascular endothelial cells. *Abbreviation*: MHC II pathway, major histocompatibility complex II pathway.

Spatial transcriptomics elucidated the pathological niches within IPF tissue. Three disease‐related niches with unique cellular compositions and locations were revealed in IPF [13]: the fibrotic niche, the airway macrophage niche, and the immune niche (Figure 6). The fibrotic niche, composed of myofibroblasts and abnormal basal cells, is located around the airways, with the highest TGF‐β signaling activity and enrichment of EMT and hypoxia pathways. The airway macrophage niche, which contains mainly SPP1^+^ macrophages, is located inside the distal airways and shows increased TNF‐α signaling activity, along with increased neutrophil activation, interferon‐γ response and IL‐6 regulation. Notably, the airway macrophage niche is adjacent to the fibrotic niche, with SPP1^+^ macrophages interacting closely with abnormal basal cells, mainly through the major histocompatibility complex II pathway. The immune niche, containing mainly B cells and T cells, serves as a histologically visible center of pathological immune cell infiltration and is colocalized with the recently identified COL15A1^+^ pVEs [10, 198]. Therefore, the immune niche is possibly recruited by the unique secretory signals of pVEs. Furthermore, spatial transcriptomics has revealed distinct niche differences between human IPF tissues and BLM‐mouse model. In patients with IPF, alveolar regeneration is impaired within fibrotic niches, whereas in the mouse model, active tissue repair is observed. Therefore, the BLM‐mouse model cannot fully recapitulate the spatial characteristics of human IPF pathology [199].

### 3.2 Discovering Cellular Trajectory and Interactions in Situ

Spatial transcriptomics also revealed and validated information on cellular trajectory and interactions within IPF tissues. Franzen et al. [199] decomposed spatial transcriptomics data into 30 “Factors” through nonnegative matrix factorization, identifying Factor14 (F14) as particularly active in IPF tissue, likely reflecting pathological remodeling. The activity of F14 is related to the density of abnormal KRT5^−^/KRT17^+^ basaloid cells, myofibroblasts, and HAS1^hi^ fibroblast subtypes. Notably, the areas with high densities of KRT5^−^/KRT17^+^ abnormal basaloid cells were located at the edge of the fibroblast lesions, confirming the proposed location of these cells [10]. The density of transitional AT2 cells peaked near the F14‐associated niche, indicating a possible differentiation spectrum from AT2 to transitional AT2 cells and then to abnormal KRT5^−^/KRT17^+^ basaloid cells. In addition, SCGB3A2^+^ secretory cells were observed near the F14‐associated niche, supporting previous findings that they may be a source of abnormal KRT5^−^/KRT17^+^ basaloid cells. Furthermore, spatial transcriptomics revealed in situ cellular interactions. Yang et al. [200] found that in IPF lungs, mural cells surround the blood vessels in tertiary lymphoid structure (TLS) regions, expressing CCR7 ligands that attract T cells into the TLS to promote plasmacyte production. Distinct IPF‐associated fibroblasts further secrete CXCL12, providing an extramedullary niche that facilitates the dispersion and accumulation of plasmacytes in fibrotic areas. The niche formed by plasmacytes and local antibodies in the fibrotic regions is closely associated with the generation of TGF‐β signaling and the progression of pulmonary fibrosis.

The integration of single‐cell sequencing technology with spatial transcriptomics has provided a comprehensive spatiotemporal map of gene regulation during the progression of IPF. These findings highlight that IPF is a multistage, heterogeneous, and progressive disease characterized by distinct alterations in cellular gene expression and functional abnormalities at different disease stages and lesion sites. Furthermore, the pathogenesis of IPF involves complex intercellular crosstalk rather than isolated cellular actions, and spatial transcriptomics offers a precise spatial context for elucidating these intricate interactions. Future research efforts should be devoted to collecting multidimensional information from the same tissue section and advancing the development of the spatial multiomics field. These data will provide a more comprehensive view of the biological mechanisms and interaction events within the tissue ecosystem.

## 4 Therapeutic Implications: From Cellular Mechanisms to Clinical Translation

Understanding the heterogeneity and dynamics of various cell types at the single‐cell level has significantly advanced insights into therapeutic targets for IPF. Numerous cell types and their key expressed molecules, which are integral to IPF pathogenesis, are proposed as promising therapeutic targets.

### 4.1 Cell‐Type‐Specific Therapeutic Targets and Strategies

Previous studies have identified numerous cellular targets with promising therapeutic potential while investigating the cellular mechanisms underlying the pathogenesis of IPF.

Macrophage‐related therapeutic targets dominate the findings derived from single‐cell‐level studies. Numerous macrophage activity‐related pathways play critical roles in fibrosis in IPF patients, and the development of novel therapeutics may benefit from targeting these pathways. The SPP1^hi^ macrophage subtype is critically involved in the pathogenesis of IPF [10, 99, 134, 137]. Elevated expression of both SPP1 and MERTK in these macrophages appears pivotal for tissue repair and fibrosis in individuals with IPF [137]. Studies have shown that targeting SPP1 can inhibit the JAK2/STAT3 signaling pathway, suppress macrophage M2 polarization, and effectively attenuate the progression of IPF [201]. Furthermore, studies have confirmed that both pirfenidone and nintedanib exert antifibrotic effects on macrophages and fibroblasts through the SPP1–AKT pathway, indicating that SPP1 is a potential target for IPF treatment [202]. In addition, targeting MERTK may also effectively deplete profibrotic macrophages and activate fibroblasts in IPF lungs [203, 204, 205]. Moreover, it participates in the interaction between Mo_AMs and fibroblasts via the ERK pathway [139], while ERK pathway inhibitors significantly alleviate pulmonary fibrosis [131, 206]. These findings indicate the therapeutic potential of targeting the MEK/ERK pathway and JAK2/STAT3 pathway in IPF patients. The CCL2/CCR2 signaling pathway is involved in macrophage recruitment and M1 polarization during IPF development. Targeting this pathway by inhibiting CCR2 in macrophages significantly alleviated pulmonary fibrosis and prolonged the survival of radiation‐induced IPF model mice [120]. Targeting specific markers or key molecules of particular macrophage subpopulations may also have therapeutic effects. For instance, several molecular compounds targeting the important macrophage subpopulation in IPF, namely, PLA2G7^+^ macrophages, have been shown to exert inhibitory effects on PLA2G7 and the potential to ameliorate fibrosis [121, 122].

Additionally, P2RX7^+^ macrophages constitute a critical subpopulation associated with fibrosis in IPF patients. ScRNA‐seq results showed an increase in P2RX7^+^ macrophages during antifibrotic treatment with nintedanib or pirfenidone, further suggesting a promising target for therapeutic innovation. Specific P2RX7 inhibitors may increase the efficacy of antifibrotic therapies [136]. CDKN2A is an important cuproptosis‐related gene that is highly expressed in IPF macrophages. The inhibition of CDKN2A in M2 macrophages using palbociclib successfully reduced the extent of pulmonary fibrosis in a BLM‐induced lung injury mouse model [27].

In addition to macrophages, other cell type‐specific therapeutic targets for IPF have been explored. ABCs in IPF exhibit dedifferentiation and profibrotic traits. A connectome analysis of scRNA‐seq data suggested that these changes can be reversed by SRC inhibition, and experiments in both 3D culture systems and xenograft models further revealed the antifibrotic effect of SRC inhibition [49, 207].

Therapeutic targets in fibrotic cells, including TSP‐1 and ANGPTL4, have also been validated. TSP‐1 promotes ER stress and fibroblast activation through CD47‐dependent ROS production, and its inhibition significantly alleviates BLM‐induced ER stress and pulmonary fibrosis in murine models [69]. Similarly, ANGPTL4 administration exacerbates BLM‐induced pulmonary fibrosis in vivo, whereas its KO reduces disease severity. These findings highlight the therapeutic potential of targeting fibroblast‐specific pathways [208].

Recent studies have revealed the importance of CD8^+^ T‐cell metabolism in IPF progression. CD8^+^ T‐cell‐mediated JAK–STAT, NF‐κB, and MAPK signaling pathways are enriched in IPF patients [168], with inhibitors of these pathways showing therapeutic promise. For example, targeting JAK/STAT signaling improves fibrosis caused by COVID‐19 and alleviates pulmonary fibrosis in rats [209, 210]. Similarly, the inhibition of NF‐κB signaling has been shown to mitigate pulmonary fibrosis [211, 212, 213]. Furthermore, the expression of CD19, a gene that is upregulated in the B cells of IPF patients, may contribute to pulmonary fibrosis through the modulation of B‐cell infiltration [68, 214]. Targeting CD19 has emerged as a potential strategy, as it can reduce the release of inflammatory mediators, suppress B‐cell activity, alleviate interstitial fibrosis and improve lung function [214, 215, 216].

### 4.2 Challenges in Clinical Translation and Patient Stratification

Previous studies have elucidated the cellular dynamics of IPF and the key molecules or pathways involved, some of which have been identified as potential therapeutic targets for IPF in preclinical trials. Interventions targeting the pathways mentioned above have produced promising results in preclinical models and may provide valuable insights for the development of new therapies and precision treatments for IPF. However, the efficacy and safety of these interventions have yet to be clinically validated. Future research should explore suitable drugs targeting these molecular pathways and conduct clinical studies to evaluate the effects of these interventions. In addition to these promising preclinical treatment targets, numerous clinical trials have been conducted to explore drugs targeting specific molecular pathways in IPF (Table 4). The activation of JAK2 and STAT3 is considered a key factor in the fibrotic process. Studies have shown that cytokines such as TGF‐β1, IL‐6, and IL‐13 activate the JAK2/STAT3 pathway, inducing the transformation of AT2 cells and fibroblasts into myofibroblasts and thereby promoting the occurrence and development of fibrosis. Additionally, SPP1 promotes M2 macrophage polarization through the activation of the JAK2/STAT3 pathway, thereby accelerating IPF progression [201]. Inhibitors of the JAK–STAT pathway, such as JSI‐124, have been shown to exert antifibrotic effects on IPF animal models [217]. Furthermore, the efficacy of jaktinib, a drug targeting this pathway, in improving lung function in IPF patients was shown in a phase II clinical trial, although further phase III trials have yet to be conducted.

**TABLE 4:** Novel therapeutic targets and study phases.

Drug/intervention	Target	Study phase	Research/trials
SPP1 inhibitors	SPP1 (macrophage polarization and profibrotic activity)	Preclinical trial	[201, 202]
FNA‐siCCR2	CCL2/CCR2 signaling pathway (macrophages)	Preclinical trial	[120]
Palbociclib	CDKN2A (M2 macrophages)	Preclinical trial	[27]
Celecoxib, darapladib	PLA2G7 (PLA2G7^high^ macrophages)	Preclinical trial	[122]
Jaktinib, JSI‐124	JAK–STAT pathway (Immune cell‐mediated fibrosis)	Phase II	ClinicalTrials.gov (NCT04312594)
BG00011	αvβ6‐mediated TGF‐β (Involved in activation of alveolar epithelial cells, macrophages, fibroblasts)	Phase IIb	[218]
Bexotegrast/PLN‐74809	αv integrin‐mediated TGF‐β activation (Epithelial cells and fibroblasts)	Phase IIa	ClinicalTrials.gov (NCT04396756); [219]
Pamrevlumab	CTGF (profibrotic effect on fibroblasts)	Phase III	ClinicalTrials.gov (Zephyrus NCT03955146); [8]
Saracatinib	Src kinase (fibroblast activation)	Phase Ib/IIa	ClinicalTrials.gov (NCT04598919); [220]

Another crucial signaling pathway involved in IPF pathogenesis is the TGF‐β signaling pathway, which acts on various cell types, including alveolar epithelial cells [24], AMs [221], and fibroblasts [222], to promote fibrosis progression. BG00011 targets the αvβ6 integrin‐mediated activation of TGF‐β in epithelial cells and has been shown to have inhibitory effects on TGF‐β‐related biomarkers in early clinical trials [223]. However, the results of phase IIb trials have indicated that BG00011 causes severe adverse reactions, suggesting the need for the development of other drugs targeting TGF‐β and epithelial cells [218]. Another drug, bexotegrast (PLN‐74809), which showed efficacy in phase IIa clinical trials, targets αvβ6 integrin expressed on damaged/activated epithelial cells and αvβ1 integrin on fibroblasts, inhibiting TGF‐β activation mediated by both cell types [219, 224]. PLN‐74809 has shown promising therapeutic effects in current studies, although its efficacy and safety need to be further validated in larger clinical trials. CTGF enhances the effects of profibrotic signals such as TGF‐β, promoting fibroblast proliferation, migration, and differentiation into myofibroblasts [225]. Pamrevlumab, an anti‐CTGF monoclonal antibody, blocks CTGF‐mediated fibroblast activation. In early phase II studies, pamrevlumab tended to delay the decline in lung function and reduce fibrosis detected using imaging in IPF patients, with overall good tolerability. However, in the subsequent phase III (ZEPHYRUS‐1) trial, pamrevlumab did not meet the primary endpoint, with no statistically significant difference in the absolute change in the FVC between the treatment and control groups at 48 weeks [8]. In IPF, increased TGF‐β signaling further activates Src/Fyn, which promotes fibroblast proliferation, migration, differentiation into myofibroblasts, and ECM synthesis [220]. Saracatinib inhibits Src/Fyn kinase activity and disrupts the downstream profibrotic pathways induced by TGF‐β. This mechanism has been confirmed in preclinical trials [220], but the clinical efficacy of saracatinib in patient populations is still under evaluation.

The translation of identified therapeutic targets for IPF into clinical practice holds significant promise. However, several challenges must be addressed to ensure the safety and efficacy of these treatments. One primary concern is the risk of off‐target effects. The complex and heterogeneous nature of IPF means that drugs targeting specific molecular pathways can inadvertently interact with other cellular pathways, leading to adverse effects. For example, targeting the SPP1–JAK2/STAT3 pathway may interfere with other essential cellular functions, potentially resulting in immune modulation or tissue damage. Moreover, many identified therapeutic targets, such as MERTK and CCR2, are not exclusive to IPF and are involved in other diseases, which can lead to systemic side effects. Another challenge is the development of resistance mechanisms. As with other chronic diseases, drug resistance may develop over time, complicating long‐term therapeutic efficacy. IPF patients, for instance, may develop resistance to antifibrotic drugs such as pirfenidone or nintedanib because of genetic mutations or adaptive mechanisms in fibrotic cells. Therefore, understanding and addressing these risks is crucial for the successful clinical application of novel therapies. Additionally, the inherent heterogeneity among IPF patients poses a major obstacle. The molecular mechanisms driving fibrosis can vary significantly across individuals, suggesting that a one‐size‐fits‐all approach is not feasible. Different signaling pathways (e.g., the TGF‐β and JAK–STAT pathways) may be predominantly altered in distinct patient subgroups, necessitating personalized treatment strategies. Furthermore, the lack of reliable biomarkers for patient stratification remains a critical barrier. Current research has yet to identify biomarkers that can predict treatment responses or help stratify patients based on the underlying molecular mechanisms of their disease. This limitation undermines the clinical applicability of discoveries using single‐cell technologies, as treatments derived from these findings may not be effective across diverse patient populations.

In summary, while the identification of novel therapeutic targets for IPF, such as SPP1, CCL2, and CDKN2A, offers promising treatment options, several critical issues must be addressed before these findings can be translated into clinical practice. The potential risks, including off‐target effects and resistance development, underscore the need for extensive clinical testing. Additionally, overcoming the translational gap associated with patient heterogeneity and the lack of reliable biomarkers for patient stratification is essential for optimizing clinical outcomes. Future research should focus on developing personalized treatment regimens, identifying predictive biomarkers, and testing novel therapies in large, diverse clinical cohorts. Ongoing efforts to refine single‐cell technologies and integrate multiomics data into clinical trials will be pivotal in accelerating the successful translation of these discoveries into effective IPF treatments.

## 5 Conclusion and Future Perspectives

ScRNA‐seq can provide detailed insights into the transcriptional features of well‐defined cell types and rare cellular subsets within the lung microenvironment, including the identification of “intermediate cell states” during disease progression. These intermediate states display novel phenotypes that arise during the disease while maintaining the core characteristics of their specific cell types [226]. The pathogenesis and progression of IPF involve multiple cellular actors, including alveolar epithelial cells, fibroblasts, immune cells, and endothelial cells, each contributing uniquely to the fibrotic process through distinct molecular markers, gene expression patterns, and functional roles. ScRNA‐seq enables the precise delineation of the roles and functional states of these diverse cell populations in the context of disease, providing a new perspective on the complex pathophysiological mechanisms underlying IPF. Notably, these cell subsets in IPF, which exhibit dysfunction and altered expression patterns, are not isolated entities. They collaboratively participate in the pathogenesis and progression of the disease. Through a range of molecular signals, these cells interact with one another, forming an epithelial–fibroblast–immune cell interaction loop that self‐sustains and progressively amplifies the fibrotic process. These insights, which cannot be obtained through conventional bulk RNA sequencing because of its ability to average signals across mixed cell types, underscore the dynamic cellular alterations in IPF and provide a theoretical foundation for targeted therapeutic strategies.

Despite these advances, conventional scRNA‐seq approaches have several technical limitations. By design, droplet‐based methods capture only a fraction of the transcripts in a cell, leading to high “dropout” rates (false‐zero measurements) for low‐abundance genes [227]. Such stochastic missingness, together with variable RNA capture and amplification efficiency, introduces substantial technical noise that can obscure true biological differences. Batch‐to‐batch variation (arising from differences in sample preparation or sequencing runs) can further complicate the interpretation unless carefully corrected [228]. Moreover, ambient RNA released from lysed cells can contaminate droplets, lowering the signal‐to‐noise ratio and potentially misassigning transcripts to the wrong cells [229]. In practice, these factors limit sensitivity and accuracy; for example, standard scRNA‐seq may underdetect transcripts in small cells or rare populations, and spurious gene expression can arise from neighboring cell debris. Such limitations call for a cautious analysis and underscore the need for complementary methods.

To overcome these challenges, emerging multimodal technologies are enhancing single‐cell studies. CITE‐seq and related techniques extend transcriptomics with proteomic information using oligonucleotide‐barcoded antibodies to quantify tens to hundreds of cell‐surface proteins in parallel with mRNA [230]. This approach allows researchers to link protein markers with transcriptional profiles, yielding a more complete cell identity and state. Spatial transcriptomics preserves the tissue context by measuring gene expression in situ; for instance, an image‐based spatial transcriptomic atlas of IPF lungs (∼1.6 million cells across 35 subjects) revealed distinct molecular niches and tracked the progression of injury from alveolar epithelial dysregulation to macrophage polarization in space [231]. Similarly, multiomic assays that coprofile RNA and chromatin (e.g., 10x Genomics Single‐Cell Multiome) now permit the simultaneous measurement of the transcriptome and open chromatin in the same cell. In IPF, combined snATAC‐seq and scRNA‐seq revealed the enrichment of TWIST1 TF motifs in fibrotic myofibroblasts, directly linking chromatin accessibility to a key regulatory program of fibrosis [100]. These and other emerging tools (e.g., single‐cell metabolomics, spatial proteomics, and single‐cell methylation) provide richer phenotypic and regulatory information, which can mitigate the limitations of RNA‐seq alone. In fact, integrative multiomics approaches have begun to be used; for example, Wen et al. [232] combined genomic (GWAS and eQTL) data with bulk, single‐cell and spatial transcriptomic profiles to identify new IPF susceptibility genes and potential drug targets.

Looking forward, the integration of multimodal single‐cell datasets promises to accelerate biomarker discovery and personalized therapy for IPF. By jointly analyzing transcriptomic, proteomic, and spatial information, researchers can identify cell type‐specific and niche‐specific molecular signatures of this disease. For instance, surface markers discovered using CITE‐seq might serve as targets for novel blood biomarkers or imaging probes, whereas chromatin or epigenetic signatures could inform patient stratification. Ultimately, patient‐tailored therapies may emerge from these atlases: integrating multiomic profiles with clinical data could reveal which fibrogenic pathways dominate in individual patients, guiding the choice of targeted interventions. In summary, while challenges remain, the convergence of single‐cell and spatial multiomics heralds a more precise and mechanistic understanding of IPF, with the potential to translate into predictive biomarkers and precision medicine strategies in the near future.

## Author Contributions

Liang Wu and Jiang Fan designed and supervised the project. Lin Zuo and Qiong‐Liang Liu collected the literatures and drafted the original manuscript. Liang Wu, Jiang Fan, and De‐Feng Ye revised the draft. All authors discussed and commented on the manuscript. All authors have read and approved the final manuscript.

## Funding

This work was supported by National Natural Science Foundation of China (8197010641).

## Ethics Statement

The authors have nothing to report.

## Conflicts of Interest

The authors declare no conflicts of interest.

## Data Availability

The authors have nothing to report.

## References

[1] L. Richeldi , H. R. Collard , and M. G. Jones , “Idiopathic Pulmonary Fibrosis,” Lancet (London, England) 389 (2017): 1941–1952. 28365056 10.1016/S0140-6736(17)30866-8

[2] F. J. Martinez , H. R. Collard , A. Pardo , et al., “Idiopathic Pulmonary Fibrosis,” Nature Reviews Disease Primers 3 (2017): 17074. 10.1038/nrdp.2017.74 29052582

[3] F. Luppi , M. Kalluri , P. Faverio , M. Kreuter , and G. Ferrara , “Idiopathic Pulmonary Fibrosis beyond the Lung: Understanding Disease Mechanisms to Improve Diagnosis and Management,” Respiratory Research 22 (2021): 109. 33865386 10.1186/s12931-021-01711-1 PMC8052779

[4] Y. Park , C. Ahn , and T. Kim , “Occupational and Environmental Risk Factors of Idiopathic Pulmonary Fibrosis: a Systematic Review and Meta‐analyses,” Scientific Reports 11 (2021): 4318. 33654111 10.1038/s41598-021-81591-z PMC7925580

[5] Q. Mei , Z. Liu , H. Zuo , Z. Yang , and J. Qu , “Idiopathic Pulmonary Fibrosis: an Update on Pathogenesis,” Frontiers in Pharmacology 12 (2022): 797292. 35126134 10.3389/fphar.2021.797292 PMC8807692

[6] J. Wang , K. Li , D. Hao , et al., “Pulmonary Fibrosis: Pathogenesis and Therapeutic Strategies,” MedComm 5 (2024): e744. 39314887 10.1002/mco2.744 PMC11417429

[7] T. E. King , W. Z. Bradford , S. Castro‐Bernardini , et al., “A Phase 3 Trial of pirfenidone in Patients with Idiopathic Pulmonary Fibrosis,” New England Journal of Medicine 370 (2014): 2083–2092. 24836312 10.1056/NEJMoa1402582

[8] G. Raghu , L. Richeldi , E. R. Fernández Pérez , et al., “Pamrevlumab for Idiopathic Pulmonary Fibrosis,” Jama 332 (2024): 380–389. 38762797 10.1001/jama.2024.8693 PMC11304118

[9] L. Richeldi , C. Schiffman , J. Behr , et al., “Zinpentraxin Alfa for Idiopathic Pulmonary Fibrosis: the Randomized Phase III STARSCAPE Trial,” American Journal of Respiratory and Critical Care Medicine 209 (2024): 1132–1140. 38354066 10.1164/rccm.202401-0116OC PMC11092957

[10] T. S. Adams , J. C. Schupp , S. Poli , et al., “Single‐cell RNA‐seq Reveals Ectopic and Aberrant Lung‐resident Cell Populations in Idiopathic Pulmonary Fibrosis,” Science Advances 6 (2020): eaba1983. 32832599 10.1126/sciadv.aba1983 PMC7439502

[11] T. Tsukui , K. Sun , J. B. Wetter , et al., “Collagen‐producing Lung Cell Atlas Identifies Multiple Subsets with Distinct Localization and Relevance to Fibrosis,” Nature Communications 11 (2020): 1920. 10.1038/s41467-020-15647-5 PMC7174390 32317643

[12] A. C. Habermann , A. J. Gutierrez , L. T. Bui , et al., “Single‐cell RNA Sequencing Reveals Profibrotic Roles of Distinct Epithelial and Mesenchymal Lineages in Pulmonary Fibrosis,” Science Advances 6 (2020): eaba1972. 32832598 10.1126/sciadv.aba1972 PMC7439444

[13] C. H. Mayr , D. Santacruz , S. Jarosch , et al., “Spatial Transcriptomic Characterization of Pathologic Niches in IPF,” Science Advances 10 (2024): eadl5473. 39121212 10.1126/sciadv.adl5473 PMC11313858

[14] J. Katzen and M. F. Beers , “Contributions of Alveolar Epithelial Cell Quality Control to Pulmonary Fibrosis,” Journal of Clinical Investigation 130 (2020): 5088–5099. 32870817 10.1172/JCI139519 PMC7524463

[15] C. Yao , X. Guan , G. Carraro , et al., “Senescence of Alveolar Type 2 Cells Drives Progressive Pulmonary Fibrosis,” American Journal of Respiratory and Critical Care Medicine 203 (2021): 707–717. 32991815 10.1164/rccm.202004-1274OC PMC7958503

[16] J. J. Gokey , J. Snowball , A. Sridharan , et al., “MEG3 is Increased in Idiopathic Pulmonary Fibrosis and Regulates Epithelial Cell Differentiation,” JCI Insight 3 (2018): e122490. 122490. 30185671 10.1172/jci.insight.122490 PMC6171798

[17] Y. Xu , T. Mizuno , A. Sridharan , et al., “Single‐cell RNA Sequencing Identifies Diverse Roles of Epithelial Cells in Idiopathic Pulmonary Fibrosis,” JCI Insight 1 (2016): e90558. 27942595 10.1172/jci.insight.90558 PMC5135277

[18] K. Y. Huang and E. Petretto , “Cross‐species Integration of Single‐cell RNA‐seq Resolved Alveolar‐epithelial Transitional States in Idiopathic Pulmonary Fibrosis,” American Journal of Physiology. Lung Cellular and Molecular Physiology 321 (2021): L491–L506. 34132117 10.1152/ajplung.00594.2020

[19] Z. Wen and A. Ablimit , “Comprehensive Analysis of scRNA‐Seq and Bulk RNA‐Seq Reveals Ubiquitin Promotes Pulmonary Fibrosis in Chronic Pulmonary Diseases,” Scientific Reports 14 (2024): 21195. 39261509 10.1038/s41598-024-70659-1 PMC11390722

[20] I. Vadász , C. H. Weiss , and J. I. Sznajder , “Ubiquitination and Proteolysis in Acute Lung Injury,” Chest 141, no. 3 (2012): 763–771. 22396561 10.1378/chest.11-1660 PMC3296459

[21] S. Guo , Y. Dong , C. Wang , et al., “Integrative Analysis Reveals the Recurrent Genetic Etiologies in Idiopathic Pulmonary Fibrosis,” QJM ‐ Monthly Journal of the Association of Physicians 116 (2023): 983–992. 37688571 10.1093/qjmed/hcad206

[22] X. Tong , G. Lin , and H. Ji , ““Dr. Jekyll and Mr. Hyde”: AT2 Cells in Lung Regeneration and Tumor Development,” Cancer Research 85 (2025): 1753–1754. 40126536 10.1158/0008-5472.CAN-25-1177

[23] T. Parimon , P. Chen , B. R. Stripp , et al., “Senescence of Alveolar Epithelial Progenitor Cells: a Critical Driver of Lung Fibrosis,” American Journal of Physiology. Cell Physiology 325 (2023): C483–C495. 37458437 10.1152/ajpcell.00239.2023 PMC10511168

[24] Y. Enomoto , H. Katsura , T. Fujimura , et al., “Autocrine TGF‐β‐positive Feedback in Profibrotic AT2‐lineage Cells Plays a Crucial Role in Non‐inflammatory Lung Fibrogenesis,” Nature Communications 14 (2023): 4956. 10.1038/s41467-023-40617-y PMC10471635 37653024

[25] L. Li , C. Yu , C. Huang , et al., “Attenuation of Ventilation‐Enhanced Epithelial‐Mesenchymal Transition through the Phosphoinositide 3‐Kinase‐γ in a Murine Bleomycin‐Induced Acute Lung Injury Model,” International Journal of Molecular Sciences 24 (2023): 5538. 36982609 10.3390/ijms24065538 PMC10053679

[26] Q. Li , Y. Wang , L. Ji , et al., “Cellular and Molecular Mechanisms of Fibrosis and Resolution in Bleomycin‐induced Pulmonary Fibrosis Mouse Model Revealed by Spatial Transcriptome Analysis,” Heliyon 9 (2023): e22461. 38125541 10.1016/j.heliyon.2023.e22461 PMC10730595

[27] B. Xu , K. Yang , X. Han , and J. Hou , “Cuproptosis‐related Gene CDKN2A as a Molecular Target for IPF Diagnosis and Therapeutics,” Inflammation Research: Official Journal of the European Histamine Research Society … [et al] 72 (2023): 1147–1160. 10.1007/s00011-023-01739-7 37166466

[28] R. M. Li , D. B. A. Tan , C. Tedja , et al., “Pre‐Treatment MMP7 Predicts Progressive Idiopathic Pulmonary Fibrosis in Antifibrotic Treated Patients,” Respirol Carlton Vic 30 (2025): 504–514. 10.1111/resp.14894 PMC12128705 39919729

[29] Z. Yang , Y. Yang , X. Han , and J. Hou , “Novel AT2 Cell Subpopulations and Diagnostic Biomarkers in IPF: Integrating Machine Learning with Single‐cell Analysis,” International Journal of Molecular Sciences 25, no. 14 (2024): 7754. 39062997 10.3390/ijms25147754 PMC11277372

[30] S. Chen , X. Zhang , C. Yang , S. Wang , and H. Shen , “Essential Role of IL‐17 in Acute Exacerbation of Pulmonary Fibrosis Induced by Non‐typeable Haemophilus influenzae,” Theranostics 12 (2022): 5125–5137. 35836804 10.7150/thno.74809 PMC9274745

[31] X. Chen , C. Shi , H. Cao , et al., “The Hedgehog and Wnt/β‐catenin System Machinery Mediate Myofibroblast Differentiation of LR‐MSCs in Pulmonary Fibrogenesis,” Cell Death & Disease 9 (2018): 639. 29844390 10.1038/s41419-018-0692-9 PMC5974360

[32] G. Huang , J. Zhang , G. Qing , et al., “S100A2 Silencing Relieves Epithelial‐Mesenchymal Transition in Pulmonary Fibrosis by Inhibiting the Wnt/β‐Catenin Signaling Pathway,” Dna and Cell Biology 40 (2021): 18–25. 33306933 10.1089/dna.2020.6030

[33] V. J. Craig , L. Zhang , J. S. Hagood , and C. A. Owen , “Matrix Metalloproteinases as Therapeutic Targets for Idiopathic Pulmonary Fibrosis,” American Journal of Respiratory Cell and Molecular Biology 53 (2015): 585–600. 26121236 10.1165/rcmb.2015-0020TR PMC4742954

[34] J. Khoury , F. Ahangari , T. Adams , et al. The role of GPR87 in Pulmonary Fibrosis. bioRxiv. Preprint at (2024), 10.1101/2024.10.10.617569.

[35] K. Heinzelmann , Q. Hu , Y. Hu , et al., “Single‐cell RNA Sequencing Identifies G‐protein Coupled Receptor 87 as a Basal Cell Marker Expressed in Distal Honeycomb Cysts in Idiopathic Pulmonary Fibrosis,” European Respiratory Journal 59 (2022): 2102373. 35604813 10.1183/13993003.02373-2021 PMC9203838

[36] S. Wang , W. Rao , A. Hoffman , et al., “Cloning a Profibrotic Stem Cell Variant in Idiopathic Pulmonary Fibrosis,” Science Translational Medicine 15 (2023): eabp9528. 37099633 10.1126/scitranslmed.abp9528 PMC10794039

[37] C. Jin , Y. Chen , Y. Wang , et al., “Single‐cell RNA Sequencing Reveals Special Basal Cells and Fibroblasts in Idiopathic Pulmonary Fibrosis,” Scientific Reports 14 (2024): 15778. 38982264 10.1038/s41598-024-66947-5 PMC11233624

[38] W. Zuo , M. R. Rostami , M. LeBlanc , et al., “Dysregulation of Club Cell Biology in Idiopathic Pulmonary Fibrosis,” PLoS ONE 15 (2020): e0237529. 32941426 10.1371/journal.pone.0237529 PMC7498242

[39] S. Wang , Y. Wang , C. Liu , et al., “EPAS1 (Endothelial PAS Domain Protein 1) Orchestrates Transactivation of Endothelial ICAM1 (Intercellular Adhesion Molecule 1) by Small Nucleolar RNA Host Gene 5 (SNHG5) to Promote Hypoxic Pulmonary Hypertension,” Hypertens Dallas Tex 1979 78, no. 4 (2021): 1080–1091. 10.1161/HYPERTENSIONAHA.121.16949 34455812

[40] K. Chang , X. Zhang , S. Lin , et al., “Atractylodin Suppresses TGF‐β‐Mediated Epithelial‐Mesenchymal Transition in Alveolar Epithelial Cells and Attenuates Bleomycin‐Induced Pulmonary Fibrosis in Mice,” International Journal of Molecular Sciences 22 (2021): 11152. 34681813 10.3390/ijms222011152 PMC8570326

[41] L. Zhu , X. Fu , X. Chen , X. Han , and P. Dong , “M2 macrophages Induce EMT through the TGF‐β/Smad2 Signaling Pathway,” Cell Biology International 41 (2017): 960–968. 28493530 10.1002/cbin.10788

[42] G. T. DiGiovanni , W. Han , T. P. Sherrill , et al., “Epithelial Yap/Taz Are Required for Functional Alveolar Regeneration Following Acute Lung Injury,” JCI Insight 8 (2023): e173374. 37676731 10.1172/jci.insight.173374 PMC10629815

[43] R. Warren , H. Lyu , K. Klinkhammer , and S. P. De Langhe , “Hippo Signaling Impairs Alveolar Epithelial Regeneration in Pulmonary Fibrosis,” Elife 12 (2023): e85092. 37166104 10.7554/eLife.85092 PMC10208641

[44] W. Zhu , C. Tan , and J. Zhang , “Aging of Alveolar Type 2 Cells Induced by Lonp1 Deficiency Exacerbates Pulmonary Fibrosis,” Biomol Biomed 24 (2024): 1258–1272. 38625722 10.17305/bb.2024.10429 PMC11378998

[45] Z. Borok , M. Horie , P. Flodby , et al., “Grp78 Loss in Epithelial Progenitors Reveals an Age‐linked Role for Endoplasmic Reticulum Stress in Pulmonary Fibrosis,” American Journal of Respiratory and Critical Care Medicine 201 (2020): 198–211. 31738079 10.1164/rccm.201902-0451OC PMC6961744

[46] X. Li , J. Wu , X. Sun , et al., “Autophagy Reprograms Alveolar Progenitor Cell Metabolism in Response to Lung Injury,” Stem Cell Reports 14 (2020): 420–432. 32059792 10.1016/j.stemcr.2020.01.008 PMC7066233

[47] G. Carraro , A. Mulay , C. Yao , et al., “Single‐Cell Reconstruction of Human Basal Cell Diversity in Normal and Idiopathic Pulmonary Fibrosis Lungs,” American Journal of Respiratory and Critical Care Medicine 202 (2020): 1540–1550. 32692579 10.1164/rccm.201904-0792OC PMC7706153

[48] E. E. Morrisey , “Basal Cells in Lung Development and Repair,” Developmental Cell 44 (2018): 653–654. 29587138 10.1016/j.devcel.2018.03.004

[49] B. Jaeger , J. C. Schupp , L. Plappert , et al., “Airway Basal Cells Show a Dedifferentiated KRT17highPhenotype and Promote Fibrosis in Idiopathic Pulmonary Fibrosis,” Nature Communications 13 (2022): 5637. 10.1038/s41467-022-33193-0 PMC9513076 36163190

[50] X. Zhang , D. Liu , Y. Hayashida , et al., “G Protein‐Coupled Receptor 87 (GPR87) Promotes Cell Proliferation in Human Bladder Cancer Cells,” International Journal of Molecular Sciences 16 (2015): 24319–24331. 26473854 10.3390/ijms161024319 PMC4632752

[51] Y. Zhang , Y. Qian , W. Lu , and X. Chen , “The G Protein‐coupled Receptor 87 Is Necessary for p53‐dependent Cell Survival in Response to Genotoxic Stress,” Cancer Research 69 (2009): 6049–6056. 19602589 10.1158/0008-5472.CAN-09-0621 PMC2719689

[52] X. Du , Z. Ma , Y. Xing , et al., “Identification and Validation of Potential Biomarkers Related to Oxidative Stress in Idiopathic Pulmonary Fibrosis,” Immunobiology 229 (2024): 152791. 39180853 10.1016/j.imbio.2024.152791

[53] T. Takashima , C. Zeng , E. Murakami , et al., “Involvement of lncRNA MIR205HG in Idiopathic Pulmonary Fibrosis and IL‐33 Regulation via Alu Elements,” JCI Insight 10, no. 5 (2025): e187172. 40059822 10.1172/jci.insight.187172 PMC11949018

[54] N. Otaki , Y. Motomura , T. Terooatea , et al., “Activation of ILC2s through Constitutive IFNγ Signaling Reduction Leads to Spontaneous Pulmonary Fibrosis,” Nature Communications 14, no. 1 (2023): 8120. 10.1038/s41467-023-43336-6 PMC10721793 38097562

[55] F. Zuo , N. Kaminski , E. Eugui , et al., “Gene Expression Analysis Reveals Matrilysin as a Key Regulator of Pulmonary Fibrosis in Mice and Humans,” PNAS 99 (2002): 6292–6297. 11983918 10.1073/pnas.092134099 PMC122942

[56] J. S. Munger , X. Huang , H. Kawakatsu , et al., “The Integrin Alpha v Beta 6 Binds and Activates Latent TGF Beta 1: a Mechanism for Regulating Pulmonary Inflammation and Fibrosis,” Cell 96 (1999): 319–328. 10025398 10.1016/s0092-8674(00)80545-0

[57] D. Lagares , P. Ghassemi‐Kakroodi , C. Tremblay , et al., “ADAM10‐mediated Ephrin‐B2 Shedding Promotes Myofibroblast Activation and Organ Fibrosis,” Nature Medicine 23 (2017): 1405–1415. 10.1038/nm.4419 PMC5720906 29058717

[58] P. Khan , K. Fytianos , S. Blumer , et al., “Basal‐Like Cell‐Conditioned Medium Exerts Anti‐Fibrotic Effects in Vitro and in Vivo,” Frontiers in Bioengineering and Biotechnology 10 (2022): 844119. 35350187 10.3389/fbioe.2022.844119 PMC8957873

[59] N. López‐Valdez , M. Rojas‐Lemus , P. Bizarro‐Nevares , et al., “The Multiple Facets of the Club Cell in the Pulmonary Epithelium,” Histology and Histopathology 39, no. 8 (2024): 969–982. 38329181 10.14670/HH-18-713

[60] T. Yokoyama , T. Yanagihara , K. Suzuki , et al., “Depletion of Club Cells Attenuates Bleomycin‐induced Lung Injury and Fibrosis in Mice,” Journal of inflammation (London, England) 14 (2017): 20. 28936122 10.1186/s12950-017-0168-1 PMC5604393

[61] J. Fukumoto , R. Soundararajan , J. Leung , et al., “The Role of Club Cell Phenoconversion and Migration in Idiopathic Pulmonary Fibrosis,” Aging 8, no. 11 (2016): 3091–3109. 27899769 10.18632/aging.101115 PMC5191887

[62] P. Sava , A. Ramanathan , A. Dobronyi , et al., “Human Pericytes Adopt Myofibroblast Properties in the Microenvironment of the IPF Lung,” JCI Insight 2, no. 24 (2017): e96352. 29263297 10.1172/jci.insight.96352 PMC5752282

[63] S.‐Y. Park , J. Y. Hong , S. Y. Lee , et al., “Club Cell‐specific Role of Programmed Cell Death 5 in Pulmonary Fibrosis,” Nature Communications 12, no. 1 (2021): 2923. 10.1038/s41467-021-23277-8 PMC8134485 34011956

[64] C. Ramos , M. Montaño , J. García‐Alvarez , et al., “Fibroblasts from Idiopathic Pulmonary Fibrosis and Normal Lungs Differ in Growth Rate, Apoptosis, and Tissue Inhibitor of Metalloproteinases Expression,” American Journal of Respiratory Cell and Molecular Biology 24 (2001): 591–598. 11350829 10.1165/ajrcmb.24.5.4333

[65] D. W. Waters , K. E. C. Blokland , P. S. Pathinayake , et al., “Fibroblast Senescence in the Pathology of Idiopathic Pulmonary Fibrosis,” American Journal of Physiology. Lung Cellular and Molecular Physiology 315 (2018): L162–L172. 29696986 10.1152/ajplung.00037.2018 PMC6139657

[66] T. Liu , F. Gonzalez De Los Santos , A. E. Rinke , C. Fang , K. R. Flaherty , and S. H. Phan , “B7H3‐dependent Myeloid‐derived Suppressor Cell Recruitment and Activation in Pulmonary Fibrosis,” Frontiers in immunology 13 (2022): 901349. 36045668 10.3389/fimmu.2022.901349 PMC9420866

[67] R. Wang and Y. Yang , “Identification of Potential Biomarkers for Idiopathic Pulmonary Fibrosis and Validation of TDO2 as a Potential Therapeutic Target,” World Journal of Cardiology 15 (2023): 293–308. 37397828 10.4330/wjc.v15.i6.293 PMC10308271

[68] L. Chen , H. Lin , L. Qin , et al., “Identification and Validation of Mutual Hub Genes in Idiopathic Pulmonary Fibrosis and Rheumatoid Arthritis‐associated Usual Interstitial Pneumonia,” Heliyon 10 (2024): e28088. 38571583 10.1016/j.heliyon.2024.e28088 PMC10987927

[69] J. Zhan , J. Wei , L. Liu , et al., “Investigation of a UPR‐related Gene Signature Identifies the Pro‐fibrotic Effects of Thrombospondin‐1 by Activating CD47/ROS/Endoplasmic Reticulum Stress Pathway in Lung Fibroblasts,” Antioxidant 12, no. 12 (2023): 2024. 10.3390/antiox12122024 PMC10740656 38136144

[70] X. Liu , M. Yang , J. Li , et al., “Identification of CFH and FHL2 as Biomarkers for Idiopathic Pulmonary Fibrosis,” Frontiers in Medicine 11 (2024): 1363643. 38784225 10.3389/fmed.2024.1363643 PMC11111937

[71] S. Vang , E. S. Helton , Y. Guo , et al., “O‐GlcNAc Transferase Regulates Collagen Deposition and Fibrosis Resolution in Idiopathic Pulmonary Fibrosis,” Frontiers in Immunology 15 (2024): 1387197. 38665916 10.3389/fimmu.2024.1387197 PMC11043510

[72] Q. Liu , Y. Bi , S. Song , et al., “Exosomal miR‐17‐5p from human Embryonic Stem Cells Prevents Pulmonary Fibrosis by Targeting Thrombospondin‐2,” Stem Cell Research & Therapy 14 (2023): 234. 37667335 10.1186/s13287-023-03449-7 PMC10478444

[73] S. Marshall , V. Bacote , and R. R. Traxinger , “Discovery of a Metabolic Pathway Mediating Glucose‐induced Desensitization of the Glucose Transport System. Role of Hexosamine Biosynthesis in the Induction of Insulin Resistance,” Journal of Biological Chemistry 266 (1991): 4706–4712. 2002019

[74] G. W. Hart , C. Slawson , G. Ramirez‐Correa , and O. Lagerlof , “Cross Talk between O‐GlcNAcylation and Phosphorylation: Roles in Signaling, Transcription, and Chronic Disease,” Annual Review of Biochemistry 80 (2011): 825–858. 10.1146/annurev-biochem-060608-102511 PMC3294376 21391816

[75] Y. Liao , X. Peng , Y. Yang , et al., “Exploring ABHD5 as a Lipid‐Related Biomarker in Idiopathic Pulmonary Fibrosis: Integrating Machine Learning, Bioinformatics, and in Vitro Experiments,” Inflammation 48, no. 3 (2025): 1176–1192., https://pubmed.ncbi.nlm.nih.gov/39046603. ISSN1573‐2576. 39046603 10.1007/s10753-024-02107-1

[76] E. F. Redente , S. Chakraborty , S. Sajuthi , et al., “Loss of Fas Signaling in Fibroblasts Impairs Homeostatic Fibrosis Resolution and Promotes Persistent Pulmonary Fibrosis,” JCI Insight 6 (2020): e141618. 141618. 33290280 10.1172/jci.insight.141618 PMC7821600

[77] C. H. Mayr , A. Sengupta , S. Asgharpour , et al., “Sfrp1 inhibits Lung Fibroblast Invasion during Transition to Injury‐induced Myofibroblasts,” European Respiratory Journal 63 (2024): 2301326. 38212077 10.1183/13993003.01326-2023 PMC10850614

[78] J. Herrera , C. Forster , T. Pengo , et al., “Registration of the Extracellular Matrix Components Constituting the Fibroblastic Focus in Idiopathic Pulmonary Fibrosis,” JCI Insight 4 (2019): e125185. 30626754 10.1172/jci.insight.125185 PMC6485370

[79] Y. Chen , Y. Sun , Y. Cui , et al., “High CTHRC1 Expression May be Closely Associated with Angiogenesis and Indicates Poor Prognosis in Lung Adenocarcinoma Patients,” Cancer Cell International 19 (2019): 318. 31798347 10.1186/s12935-019-1041-5 PMC6884781

[80] W. He , H. Zhang , Y. Wang , et al., “CTHRC1 induces Non‐small Cell Lung Cancer (NSCLC) Invasion through Upregulating MMP‐7/MMP‐9,” BMC Cancer 18 (2018): 400. 29631554 10.1186/s12885-018-4317-6 PMC5891957

[81] C. S. Trempus , B. N. Papas , M. I. Sifre , et al., “Functional Pdgfra Fibroblast Heterogeneity in Normal and Fibrotic Mouse Lung,” JCI Insight 8 (2023): e164380. 37824216 10.1172/jci.insight.164380 PMC10721331

[82] T. Xie , Y. Wang , N. Deng , et al., “Single‐Cell Deconvolution of Fibroblast Heterogeneity in Mouse Pulmonary Fibrosis,” Cell Reports 22 (2018): 3625–3640. 29590628 10.1016/j.celrep.2018.03.010 PMC5908225

[83] X. Liu , S. C. Rowan , J. Liang , et al., “Categorization of Lung Mesenchymal Cells in Development and Fibrosis,” iScience 24 (2021): 102551. 34151224 10.1016/j.isci.2021.102551 PMC8188567

[84] S. Monkley , C. Overed‐Sayer , H. Parfrey , et al., “Sensitization of the UPR by Loss of PPP1R15A Promotes Fibrosis and Senescence in IPF,” Scientific Reports 11 (2021): 21584. 34732748 10.1038/s41598-021-00769-7 PMC8566588

[85] M. J. Schafer , T. A. White , K. Iijima , et al., “Cellular Senescence Mediates Fibrotic Pulmonary Disease,” Nature Communications 8 (2017): 14532. 10.1038/ncomms14532 PMC5331226 28230051

[86] A. Venosa , “Senescence in Pulmonary Fibrosis: between Aging and Exposure,” Frontiers in Medicine 7 (2020): 606462. 33282895 10.3389/fmed.2020.606462 PMC7689159

[87] J. Zhao , C. Jing , R. Fan , and W. Zhang , “Prognostic Model of Fibroblasts in Idiopathic Pulmonary Fibrosis by Combined Bulk and Single‐cell RNA‐sequencing,” Heliyon 10, no. 14 (2024): E34519. 39113997 10.1016/j.heliyon.2024.e34519 PMC11305307

[88] J. Hou , Y. Yang , and X. Han , “Machine Learning and Single‐Cell Analysis Identify Molecular Features of IPF‐Associated Fibroblast Subtypes and Their Implications on IPF Prognosis,” International Journal of Molecular Sciences 25 (2023): 94. 38203265 10.3390/ijms25010094 PMC10778894

[89] M. Jia , L. Rosas , M. G. Kapetanaki , et al., “Early Events Marking Lung Fibroblast Transition to Profibrotic state in Idiopathic Pulmonary Fibrosis,” Respiratory Research 24 (2023): 116. 37085855 10.1186/s12931-023-02419-0 PMC10122312

[90] Y. Nakahara , N. Hashimoto , K. Sakamoto , et al., “Fibroblasts Positive for meflin Have Anti‐fibrotic Properties in Pulmonary Fibrosis,” European Respiratory Journal 58 (2021): 2003397. 34049947 10.1183/13993003.03397-2020

[91] L. Rittié , “Another Dimension to the Importance of the Extracellular Matrix in Fibrosis,” Journal of Cell Communication and Signaling 9 (2015): 99–100. 25698664 10.1007/s12079-015-0282-x PMC4414843

[92] P. Bitterman , “Fibroblast‐Matrix Cross‐Talk in Idiopathic Pulmonary Fibrosis: Cross‐Links at the Crossroads,” American Journal of Respiratory Cell and Molecular Biology 58 (2018): 547–548. 29714627 10.1165/rcmb.2017-0402ED PMC5946335

[93] M. W. Parker , D. Rossi , M. Peterson , et al., “Fibrotic Extracellular Matrix Activates a Profibrotic Positive Feedback Loop,” Journal of Clinical Investigation 124 (2014): 1622–1635. 24590289 10.1172/JCI71386 PMC3971953

[94] L. Cushing , P. P. Kuang , J. Qian , et al., “miR‐29 Is a Major Regulator of Genes Associated with Pulmonary Fibrosis,” American Journal of Respiratory Cell and Molecular Biology 45 (2011): 287–294. 20971881 10.1165/rcmb.2010-0323OC PMC3175558

[95] F. Liu , D. Lagares , K. M. Choi , et al., “Mechanosignaling through YAP and TAZ Drives Fibroblast Activation and Fibrosis,” American Journal of Physiology. Lung Cellular and Molecular Physiology 308 (2015): L344–357. 25502501 10.1152/ajplung.00300.2014 PMC4329470

[96] A. Radwanska , C. T. Cottage , A. Piras , et al., “Increased Expression and Accumulation of GDF15 in IPF Extracellular Matrix Contribute to Fibrosis,” JCI Insight 7 (2022): e153058. 35993367 10.1172/jci.insight.153058 PMC9462497

[97] F. Calabrese , F. Lunardi , V. Tauro , et al., “RNA Sequencing of Epithelial Cell/Fibroblastic Foci Sandwich in Idiopathic Pulmonary Fibrosis: New Insights on the Signaling Pathway,” International Journal of Molecular Sciences 23 (2022): 3323. 35328744 10.3390/ijms23063323 PMC8954546

[98] Y. Huang , R. Guzy , S. Ma , et al., “Central Lung Gene Expression Associates with Myofibroblast Features in Idiopathic Pulmonary Fibrosis,” BMJ Open Respiratory Research 10 (2023): e001391. 10.1136/bmjresp-2022-001391 PMC9896241 36725082

[99] P. A. Reyfman , J. M. Walter , N. Joshi , et al., “Single‐Cell Transcriptomic Analysis of Human Lung Provides Insights into the Pathobiology of Pulmonary Fibrosis,” American Journal of Respiratory and Critical Care Medicine 199 (2019): 1517–1536. 30554520 10.1164/rccm.201712-2410OC PMC6580683

[100] E. Valenzi , H. Bahudhanapati , J. Tan , et al., “Single‐nucleus Chromatin Accessibility Identifies a Critical Role for TWIST1 in Idiopathic Pulmonary Fibrosis Myofibroblast Activity,” European Respiratory Journal 62 (2023): 2200474. 37142338 10.1183/13993003.00474-2022 PMC10411550

[101] G. Epstein Shochet , E. Brook , L. Israeli‐Shani , E. Edelstein , and D. Shitrit , “Fibroblast Paracrine TNF‐α Signaling Elevates Integrin A5 Expression in Idiopathic Pulmonary Fibrosis (IPF),” Respiratory Research 18 (2017): 122. 28629363 10.1186/s12931-017-0606-x PMC5477311

[102] Y. Nie , S. Wu , Y. Xuan , and G. Yan , “Role of IL‐17 family Cytokines in the Progression of IPF from Inflammation to Fibrosis,” Military Medical Research 9 (2022): 21. 35550651 10.1186/s40779-022-00382-3 PMC9102601

[103] J. Wang , K. Hu , X. Cai , et al., “Targeting PI3K/AKT Signaling for Treatment of Idiopathic Pulmonary Fibrosis,” Acta Pharmaceutica Sinica B 12 (2022): 18–32. 35127370 10.1016/j.apsb.2021.07.023 PMC8799876

[104] J. Li , R. Yang , Y. Dong , M. Chen , Y. Wang , and G. Wang , “Correction to: Knockdown of FOXO3a Induces Epithelial‐mesenchymal Transition and Promotes Metastasis of Pancreatic Ductal Adenocarcinoma by Activation of the β‐catenin/TCF4 Pathway through SPRY2,” Journal of Experimental & Clinical Cancer Research 40 (2021): 249. 34372904 10.1186/s13046-021-02033-2 PMC8351086

[105] Y. Song , C. Miao , and J. Wang , “LncRNA ZEB1‐AS1 Inhibits Renal Fibrosis in Diabetic Nephropathy by Regulating the miR‐217/MAFB Axis,” RSC Advances 9 (2019): 30389–30397. 35557748 10.1039/c9ra05602e PMC9088285

[106] S. Xiong , C. E. Whitehurst , L. Li , et al., “Reverse Translation Approach Generates a Signature of Penetrating Fibrosis in Crohn's disease That Is Associated with Anti‐TNF Response,” Gut 71 (2022): 1289–1301. 34261752 10.1136/gutjnl-2020-323405

[107] S. Bolourani , E. Sari , M. Brenner , and P. Wang , “Extracellular CIRP Induces an Inflammatory Phenotype in Pulmonary Fibroblasts via TLR4,” Frontiers in immunology 12 (2021): 721970. 34367191 10.3389/fimmu.2021.721970 PMC8342891

[108] G. Jia , S. Chandriani , A. R. Abbas , et al., “CXCL14 is a Candidate Biomarker for Hedgehog Signalling in Idiopathic Pulmonary Fibrosis,” Thorax 72 (2017): 780–787. 28250200 10.1136/thoraxjnl-2015-207682

[109] E. Fraser , K. Blirando , V. StNoble , et al., “S50 Monocytes from IPF Patients Show Pre‐conditioned Pro‐repair Features,” Thorax 71 (2016): A30.2–A31.

[110] E. Fraser , L. Denney , C. Vuppusetty , et al., “Immunologically ‘Supercharged’ monocytes as Drivers of Chronic Fibrosis in Idiopathic Pulmonary Fibrosis,” European Respiratory Journal 56, no. suppl 64 (2020): 819.

[111] T. Kapellos , V. Viteri , D. Sirokha , et al., “Myeloid‐dependent Immunosuppressive Features Drive IPF Progression,” European Respiratory Journal 62, no. suppl 67 (2023): PA3937.

[112] A. Tzouvelekis , T. M. Maher , N. Goh , et al., “Monocyte Count and Decline in Forced Vital Capacity (FVC) in Patients with IPF,” European Respiratory Journal 56, no. suppl 64 (2020): 721.

[113] E. Fraser , L. Denney , A. Antanaviciute , et al., “Multi‐Modal Characterization of Monocytes in Idiopathic Pulmonary Fibrosis Reveals a Primed Type I Interferon Immune Phenotype,” Frontiers in Immunology 12 (2021): 623430. 33746960 10.3389/fimmu.2021.623430 PMC7973086

[114] Q. Yu , Y. Katlinskaya , C. Carbone , et al., “DNA‐damage‐induced Type I Interferon Promotes Senescence and Inhibits Stem Cell Function,” Cell Reports 11 (2015): 785–797. 25921537 10.1016/j.celrep.2015.03.069 PMC4426031

[115] T. Karampitsakos , B. Tourki , M. Jia , et al., “The Transcriptome of CD14 + CD163 ‐ HLA‐DR Low Monocytes Predicts Mortality in Idiopathic Pulmonary Fibrosis,” MedRxiv Prepr Serv Health Sci (2024), 10.1101/2024.08.07.24311386. 2024.08.07.24311386. PMC13150356 41360505

[116] E. M. Melo , V. L. S. Oliveira , D. Boff , and I. Galvão , “Pulmonary Macrophages and Their Different Roles in Health and Disease,” International Journal of Biochemistry & Cell Biology 141 (2021): 106095. 34653619 10.1016/j.biocel.2021.106095

[117] L. Deng , Z. Jian , T. Xu , et al., “Macrophage Polarization: an Important Candidate Regulator for Lung Diseases,” Mol Basel Switz 28, no. 5 (2023): 2379. 10.3390/molecules28052379 PMC10005642 36903624

[118] Z. Ge , Y. Chen , L. Ma , F. Hu , and L. Xie , “Macrophage Polarization and Its Impact on Idiopathic Pulmonary Fibrosis,” Frontiers in Immunology 15 (2024): 1444964. 39131154 10.3389/fimmu.2024.1444964 PMC11310026

[119] Y. Wang , C. Liu , Y. Xie , and X. Li , “Down‐regulation of CYTL1 Attenuates Bleomycin‐induced Pulmonary Fibrosis in Mice by Inhibiting M2 Macrophage Polarization via the TGF‐β/CCN2 Axis,” Clinical and Experimental Pharmacology & Physiology 51 (2024): e13913. 39103233 10.1111/1440-1681.13913

[120] C. Li , X. Feng , S. Li , et al., “Tetrahedral DNA Loaded siCCR2 Restrains M1 Macrophage Polarization to Ameliorate Pulmonary Fibrosis in Chemoradiation‐induced Murine Model,” Molecular Therapy is the official journal of the American Society of Gene Therapy 32 (2024): 766–782. 10.1016/j.ymthe.2024.01.022 PMC10928155 38273656

[121] J. Wang , M. Jiang , A. Xiong , et al., “Integrated Analysis of Single‐cell and Bulk RNA Sequencing Reveals Pro‐fibrotic PLA2G7high Macrophages in Pulmonary Fibrosis,” Pharmacological Research 182 (2022): 106286. 35662628 10.1016/j.phrs.2022.106286

[122] T. Liu , J. Ning , X. Fan , H. Wei , G. Shi , and Q. B. Fu , “Identification of Immune Patterns in Idiopathic Pulmonary Fibrosis Patients Driven by PLA2G7‐positive Macrophages Using an Integrated Machine Learning Survival Framework,” Scientific Reports 14 (2024): 22369. 39333367 10.1038/s41598-024-73625-z PMC11437001

[123] M. Zhu , Y. Yi , K. Jiang , et al., “Single‐cell Combined with Transcriptome Sequencing to Explore the Molecular Mechanism of Cell Communication in Idiopathic Pulmonary Fibrosis,” Journal of Cellular and Molecular Medicine 28, no. 12 (2024): e18499. ISSN 1582–1838 1582–4934. 38887981 10.1111/jcmm.18499 PMC11184282

[124] Y. Wang , C. Wu , J. Zhou , H. Fang , and J. Wang , “Overexpression of Estrogen Receptor β Inhibits Cellular Functions of human Hepatic Stellate Cells and Promotes the Anti‐fibrosis Effect of Calycosin via Inhibiting STAT3 Phosphorylation,” BMC Pharmacology and Toxicology 23 (2022): 77. 36207725 10.1186/s40360-022-00617-y PMC9541055

[125] Y. Li , G. Fang , W. Cao , et al., “Ezh2 Inhibits Replicative Senescence of Atrial Fibroblasts through Promotion of H3K27me3 in the Promoter Regions of CDKN2a and Timp4 Genes,” Journal of Inflammation Research 15 (2022): 4693–4708. 35996686 10.2147/JIR.S374951 PMC9392478

[126] P. Tsvetkov , S. Coy , B. Petrova , et al., “Copper Induces Cell Death by Targeting Lipoylated TCA Cycle Proteins,” Science 375 (2022): 1254–1261. 35298263 10.1126/science.abf0529 PMC9273333

[127] Y. Wang , L. Zhang , and F. Zhou , “Cuproptosis: a New Form of Programmed Cell Death,” Cellular & Molecular Immunology 19 (2022): 867–868. 35459854 10.1038/s41423-022-00866-1 PMC9338229

[128] Y. Kim , Y. Kim , H. J. Lim , D. Kim , J. Park , and C. Oh , “Integrative Single‐cell Transcriptome Analysis Provides New Insights into Post‐COVID‐19 Pulmonary Fibrosis and Potential Therapeutic Targets,” Journal of Medical Virology 95 (2023): e29201. 37966390 10.1002/jmv.29201

[129] A. Viola , F. Munari , R. Sánchez‐Rodríguez , T. Scolaro , and A. Castegna , “The Metabolic Signature of Macrophage Responses,” Frontiers in Immunology 10 (2019): 1462. 31333642 10.3389/fimmu.2019.01462 PMC6618143

[130] G. Soto‐Heredero , M. M. Gómez de las Heras , E. Gabandé‐Rodríguez , J. Oller , and M. Mittelbrunn , “Glycolysis—A Key Player in the Inflammatory Response,” The FEBS Journal 287, no. 16 (2020): 3350–3369. 32255251 10.1111/febs.15327 PMC7496292

[131] J. Li , X. Zhai , X. Sun , S. Cao , Q. Yuan , and J. Wang , “Metabolic Reprogramming of Pulmonary Fibrosis,” Frontiers in Pharmacology 13 (2022): 1031890. 36452229 10.3389/fphar.2022.1031890 PMC9702072

[132] M. Korfei , D. von der Beck , I. Henneke , et al., “Comparative Proteome Analysis of Lung Tissue from Patients with Idiopathic Pulmonary Fibrosis (IPF), Non‐specific Interstitial Pneumonia (NSIP) and Organ Donors,” Journal of Proteomics 85 (2013): 109–128. 23659799 10.1016/j.jprot.2013.04.033

[133] C. He and A. B. Carter , “C(C)Learing the Role of Chemokines in Pulmonary Fibrosis,” American Journal of Respiratory Cell and Molecular Biology 62 (2020): 546–547. 31951476 10.1165/rcmb.2020-0017ED PMC7193789

[134] H. Zhang , Y. Yang , Y. Cao , and J. Guan , “IPF‐related New Macrophage Subpopulations and Diagnostic Biomarker Identification—Combine Machine Learning with Single‐cell Analysis,” Respiratory Research 25 (2024): 241. 38872139 10.1186/s12931-024-02845-8 PMC11170785

[135] N. Riteau , P. Gasse , L. Fauconnier , et al., “Extracellular ATP Is a Danger Signal Activating P2×7 Receptor in Lung Inflammation and Fibrosis,” American Journal of Respiratory and Critical Care Medicine 182 (2010): 774–783. 20522787 10.1164/rccm.201003-0359OC

[136] C. Colarusso , A. Falanga , S. Di Caprio , et al., “ATP‐induced Fibrogenic Pathway in Circulating Cells Obtained by Idiopathic Pulmonary Fibrotic (IPF) Patients Is Not Blocked by nintedanib and Pirfenidone,” Biomed Pharmacother Biomedecine Pharmacother 176 (2024): 116896. 10.1016/j.biopha.2024.116896 38876049

[137] C. Morse , T. Tabib , J. Sembrat , et al., “Proliferating SPP1/MERTK‐expressing Macrophages in Idiopathic Pulmonary Fibrosis,” European Respiratory Journal 54 (2019): 1802441. 31221805 10.1183/13993003.02441-2018 PMC8025672

[138] T. Chen , J. Guo , L. Ai , et al., “Up‐regulated SPP1 Increases the Risk from IPF to Lung Cancer via Activating the Pro‐tumor Macrophages,” Computational and Structural Biotechnology Journal 21 (2023): 5751–5764. 38074471 10.1016/j.csbj.2023.11.018 PMC10708992

[139] M. Zhang , J. Zhang , H. Hu , et al., “Multiomic Analysis of Monocyte‐derived Alveolar Macrophages in Idiopathic Pulmonary Fibrosis,” Journal of Translational Medicine 22 (2024): 598. 38937806 10.1186/s12967-024-05398-y PMC11209973

[140] R. S. Scott , E. J. McMahon , S. M. Pop , et al., “Phagocytosis and Clearance of Apoptotic Cells Is Mediated by MER,” Nature 411 (2001): 207–211. 11346799 10.1038/35075603

[141] J. H. M. van der Meer , T. van der Poll , and C. Van't Veer , “TAM Receptors, Gas6, and Protein S: Roles in Inflammation and Hemostasis,” Blood 123 (2014): 2460–2469. 24596417 10.1182/blood-2013-09-528752

[142] S. Sather , K. D. Kenyon , J. B. Lefkowitz , et al., “A Soluble Form of the Mer Receptor Tyrosine Kinase Inhibits Macrophage Clearance of Apoptotic Cells and Platelet Aggregation,” Blood 109 (2007): 1026–1033. 17047157 10.1182/blood-2006-05-021634 PMC1785151

[143] H. M. Seitz , T. D. Camenisch , G. Lemke , H. S. Earp , and G. K. Matsushima , “Macrophages and Dendritic Cells Use Different Axl/Mertk/Tyro3 Receptors in Clearance of Apoptotic Cells,” Journal of immunology (Baltimore, Md: 1950) 178 (2007): 5635–5642. 17442946 10.4049/jimmunol.178.9.5635

[144] V. J. Thannickal , “Evolving Concepts of Apoptosis in Idiopathic Pulmonary Fibrosis,” Proceedings of the American Thoracic Society 3 (2006): 350–356. 16738200 10.1513/pats.200601-001TK PMC2231523

[145] M. S. Espindola , D. M. Habiel , R. Narayanan , et al., “Targeting of TAM Receptors Ameliorates Fibrotic Mechanisms in Idiopathic Pulmonary Fibrosis,” American Journal of Respiratory and Critical Care Medicine 197 (2018): 1443–1456. 29634284 10.1164/rccm.201707-1519OC PMC6005556

[146] Y. Liao , X. Peng , Y. Yang , et al., “Integrating Cellular Experiments, Single‐cell Sequencing, and Machine Learning to Identify Endoplasmic Reticulum Stress Biomarkers in Idiopathic Pulmonary Fibrosis,” Annals of Medicine 56 (2024): 2409352. 39340293 10.1080/07853890.2024.2409352 PMC11441044

[147] H. Cui , D. Jiang , S. Banerjee , et al., “Monocyte‐derived Alveolar Macrophage Apolipoprotein E Participates in Pulmonary Fibrosis Resolution,” JCI Insight 5 (2020): e134539. 134539. 32027623 10.1172/jci.insight.134539 PMC7141408

[148] R. Schierwagen , L. Maybüchen , S. Zimmer , et al., “Seven Weeks of Western Diet in Apolipoprotein‐E‐deficient Mice Induce Metabolic Syndrome and Non‐alcoholic Steatohepatitis with Liver Fibrosis,” Scientific Reports 5 (2015): 12931. 26263022 10.1038/srep12931 PMC4531783

[149] E. C. Vasquez , V. A. Peotta , A. L. Gava , T. M. Pereira , and S. S. Meyrelles , “Cardiac and Vascular Phenotypes in the Apolipoprotein E‐deficient Mouse,” Journal of Biomedical Science 19 (2012): 22. 22330242 10.1186/1423-0127-19-22 PMC3306747

[150] A. V. Misharin , L. Morales‐Nebreda , P. A. Reyfman , et al., “Monocyte‐derived Alveolar Macrophages Drive Lung Fibrosis and Persist in the Lung over the Life Span,” Journal of Experimental Medicine 214 (2017): 2387–2404. 28694385 10.1084/jem.20162152 PMC5551573

[151] E. A. Ayaub , S. Poli , J. Ng , et al. Single Cell RNA‐seq and Mass Cytometry Reveals a Novel and a Targetable Population of Macrophages in Idiopathic Pulmonary Fibrosis. bioRxiv. (2021). Preprint at, 10.1101/2021.01.04.425268.

[152] S. H. Apte , P. L. Groves , M. E. Tan , et al., “A Methodological Approach to Identify Natural Compounds with Antifibrotic Activity and the Potential to Treat Pulmonary Fibrosis Using Single‐Cell Sequencing and Primary Human Lung Macrophages,” International Journal of Molecular Sciences 24 (2023): 15104. 37894784 10.3390/ijms242015104 PMC10606775

[153] L. Wang , Z. Li , R. Wan , et al., “Single‐Cell RNA Sequencing Provides New Insights into Therapeutic Roles of Thyroid Hormone in Idiopathic Pulmonary Fibrosis,” American Journal of Respiratory Cell and Molecular Biology 69 (2023): 456–469. 37402274 10.1165/rcmb.2023-0080OC PMC10557923

[154] D. Aran , A. P. Looney , L. Liu , et al., “Reference‐based Analysis of Lung Single‐cell Sequencing Reveals a Transitional Profibrotic Macrophage,” Nature Immunology 20 (2019): 163–172. 30643263 10.1038/s41590-018-0276-y PMC6340744

[155] A. Vallée , Y. Lecarpentier , R. Guillevin , and J. Vallée , “Interactions between TGF‐β1, Canonical WNT/β‐catenin Pathway and PPAR γ in Radiation‐induced Fibrosis,” Oncotarget 8 (2017): 90579–90604. 29163854 10.18632/oncotarget.21234 PMC5685775

[156] L. Leng , C. N. Metz , Y. Fang , et al., “MIF Signal Transduction Initiated by Binding to CD74,” Journal of Experimental Medicine 197 (2003): 1467–1476. 12782713 10.1084/jem.20030286 PMC2193907

[157] Y. Lv , J. Yang , A. Gao , et al., “Sortilin Promotes Macrophage Cholesterol Accumulation and Aortic Atherosclerosis through Lysosomal Degradation of ATP‐binding Cassette Transporter A1 Protein,” Acta Biochimica et Biophysica Sinica 51 (2019): 471–483. 30950489 10.1093/abbs/gmz029

[158] Y. Chen , S. Zhu , T. Liu , et al., “Epithelial Cells Activate Fibroblasts to Promote Esophageal Cancer Development,” Cancer Cell 41 (2023): 903–918.e8. 36963399 10.1016/j.ccell.2023.03.001

[159] S. McArthur , G. Juban , T. Gobbetti , et al., “Annexin A1 Drives Macrophage Skewing to Accelerate Muscle Regeneration through AMPK Activation,” Journal of Clinical Investigation 130 (2020): 1156–1167. 32015229 10.1172/JCI124635 PMC7269594

[160] Y. Guo , Z. He , Z. Chen , et al., “Inhibition of Th17 Cells by donepezil Ameliorates Experimental Lung Fibrosis and Pulmonary Hypertension,” Theranostics 13 (2023): 1826–1842. 37064881 10.7150/thno.82069 PMC10091879

[161] C. Tao , H. Xian , Z. Nian‐yu , S. Jia‐cui , W. Dong , and L. Hui‐ping , “C‐type Lectin Mincle Initiates IL‐17‐mediated Inflammation in Acute Exacerbations of Idiopathic Pulmonary Fibrosis,” Biomed Pharmacother Biomedecine Pharmacother 159 (2023): 114253. 10.1016/j.biopha.2023.114253 36680813

[162] S. Park , H. Hahn , S. Oh , and H. Lee , “Theophylline Attenuates BLM‐Induced Pulmonary Fibrosis by Inhibiting Th17 Differentiation,” International Journal of Molecular Sciences 24 (2023): 1019. 36674533 10.3390/ijms24021019 PMC9860752

[163] S. Park , H. W. Ryu , J. Kim , et al., “Daphnetin Alleviates Bleomycin‐Induced Pulmonary Fibrosis through Inhibition of Epithelial‐to‐Mesenchymal Transition and IL‐17A,” Cells 12 (2023): 2795. 38132116 10.3390/cells12242795 PMC10742308

[164] I. Kotsianidis , E. Nakou , I. Bouchliou , et al., “Global Impairment of CD4+CD25+FOXP3+ Regulatory T Cells in Idiopathic Pulmonary Fibrosis,” American Journal of Respiratory and Critical Care Medicine 179 (2009): 1121–1130. 19342412 10.1164/rccm.200812-1936OC

[165] A. Unterman , A. Y. Zhao , N. Neumark , et al., “Single‐Cell Profiling Reveals Immune Aberrations in Progressive Idiopathic Pulmonary Fibrosis,” American Journal of Respiratory and Critical Care Medicine 210 (2024): 484–496. 38717443 10.1164/rccm.202306-0979OC PMC11351796

[166] Z. Daniil , P. Kitsanta , G. Kapotsis , et al., “CD8+ T Lymphocytes in Lung Tissue from Patients with Idiopathic Pulmonary Fibrosis,” Respiratory Research 6 (2005): 81. 16042790 10.1186/1465-9921-6-81 PMC1199622

[167] D. M. Habiel , M. S. Espindola , C. Kitson , et al., “Characterization of CD28null T Cells in Idiopathic Pulmonary Fibrosis,” Mucosal Immunol 12 (2019): 212–222. 30315241 10.1038/s41385-018-0082-8 PMC6301115

[168] X. Wei , C. Jin , D. Li , et al., “Single‐cell Transcriptomics Reveals CD8+ T Cell Structure and Developmental Trajectories in Idiopathic Pulmonary Fibrosis,” Molecular Immunology 172 (2024): 85–95. 38936318 10.1016/j.molimm.2024.06.008

[169] N. Singh , J. Perazzelli , S. A. Grupp , and D. M. Barrett , “Early Memory Phenotypes Drive T Cell Proliferation in Patients with Pediatric Malignancies,” Science Translational Medicine 8 (2016): 320ra3. 10.1126/scitranslmed.aad5222 26738796

[170] R. Parsanathan and S. K. Jain , “G6PD deficiency Shifts Polarization of Monocytes/Macrophages towards a Proinflammatory and Profibrotic Phenotype,” Cellular & Molecular Immunology 18 (2021): 770–772. 32523113 10.1038/s41423-020-0428-5 PMC8027810

[171] K. Chen , H. Hsu , C. Lee , et al., “The AMPK Agonist AICAR Inhibits TGF‐β1 Induced Activation of Kidney Myofibroblasts,” PLoS ONE 9 (2014): e106554. 25188319 10.1371/journal.pone.0106554 PMC4154690

[172] H. Zhu , Y. Chai , D. Dong , et al., “AICAR‐Induced AMPK Activation Inhibits the Noncanonical NF‐κB Pathway to Attenuate Liver Injury and Fibrosis in BDL Rats,” Canadian Journal of Gastroenterology & Hepatology 2018 (2018): 6181432. 30662889 10.1155/2018/6181432 PMC6314002

[173] M. A. Alshawsh , A. Alsalahi , S. A. Alshehade , et al., “A Comparison of the Gene Expression Profiles of Non‐Alcoholic Fatty Liver Disease between Animal Models of a High‐Fat Diet and Methionine‐Choline‐Deficient Diet,” Molecules (Basel, Switzerland) 27, no. 3 (2022): 858. 35164140 10.3390/molecules27030858 PMC8839835

[174] D. A. Pociask , K. Chen , S. Mi Choi , T. D. Oury , C. Steele , and J. K. Kolls , “γδ T Cells Attenuate Bleomycin‐induced Fibrosis through the Production of CXCL10,” American Journal of Pathology 178 (2011): 1167–1176. 21356368 10.1016/j.ajpath.2010.11.055 PMC3070585

[175] D. Okuno , N. Sakamoto , Y. Akiyama , et al., “Two Distinct Mechanisms Underlying Γδ T Cell‐Mediated Regulation of Collagen Type I in Lung Fibroblasts,” Cells 11 (2022): 2816. 36139391 10.3390/cells11182816 PMC9496746

[176] C. L. Vigeland , S. L. Collins , Y. Chan‐Li , et al., “Deletion of mTORC1 Activity in CD4+ T Cells Is Associated with Lung Fibrosis and Increased Γδ T Cells,” PLoS ONE 11 (2016): e0163288. 27649073 10.1371/journal.pone.0163288 PMC5029914

[177] P. C. Carré , R. L. Mortenson , T. E. King Jr , P. W. Noble , C. L. Sable , and D. W. Riches , “Increased Expression of the Interleukin‐8 Gene by Alveolar Macrophages in Idiopathic Pulmonary Fibrosis. A Potential Mechanism for the Recruitment and Activation of Neutrophils in Lung Fibrosis,” Journal of Clinical Investigation 88, no. 6 (1991): 1802–1810. 1752942 10.1172/JCI115501 PMC295747

[178] Y. Obayashi , I. Yamadori , J. Fujita , T. Yoshinouchi , N. Ueda , and J. Takahara , “The Role of Neutrophils in the Pathogenesis of Idiopathic Pulmonary Fibrosis,” Chest 112 (1997): 1338–1343. 9367478 10.1378/chest.112.5.1338

[179] Y. Lin , X. Lai , T. Lei , et al., “Neutrophil‐Related Gene Expression Signatures in Idiopathic Pulmonary Fibrosis: Implications for Disease Characteristic and Identification of Diagnostic Hub Genes,” Journal of Inflammation Research 16 (2023): 2503–2519. 37337515 10.2147/JIR.S414734 PMC10277023

[180] L. E. Crowley , R. A. Stockley , D. R. Thickett , D. Dosanjh , A. Scott , and D. Parekh , “Neutrophil Dynamics in Pulmonary Fibrosis: Pathophysiological and Therapeutic Perspectives,” European Respiratory Review: An Official Journal of the European Respiratory Society 33, no. 174 (2024): 240139. 39603661 10.1183/16000617.0139-2024 PMC11600124

[181] K. M. Lodge , S. Nakanishi , L. Yazbeck , J. Guck , P. L. Molyneaux , and A. S. Cowburn , “S96 Circulating Neutrophils in Idiopathic Pulmonary Fibrosis Have a Distinct Biomechanical Phenotype of Systemic Activation That Correlates with Disease Severity,” Thorax 79 (2024): A70–A71.

[182] M. Wygrecka , B. K. Dahal , D. Kosanovic , et al., “Mast Cells and Fibroblasts Work in Concert to Aggravate Pulmonary Fibrosis: Role of Transmembrane SCF and the PAR‐2/PKC‐α/Raf‐1/p44/42 Signaling Pathway,” American Journal of Pathology 182 (2013): 2094–2108. 23562441 10.1016/j.ajpath.2013.02.013

[183] A. Veerappan , N. J. O'Connor , J. Brazin , et al., “Mast Cells: a Pivotal Role in Pulmonary Fibrosis,” Dna and Cell Biology 32 (2013): 206–218. 23570576 10.1089/dna.2013.2005 PMC3624698

[184] G. Ishikawa , A. Liu , and E. L. Herzog , “Evolving Perspectives on Innate Immune Mechanisms of IPF,” Frontiers in Molecular Biosciences 8 (2021): 676569. 34434962 10.3389/fmolb.2021.676569 PMC8381017

[185] T. Planté‐Bordeneuve , C. Pilette , and A. Froidure , “The Epithelial‐Immune Crosstalk in Pulmonary Fibrosis,” Frontiers in Immunology 12 (2021): 631235. 34093523 10.3389/fimmu.2021.631235 PMC8170303

[186] M. Yoshida , R. Arzili , and M. Z. Nikolić , “Immune‐epithelial Cell Interactions in Lung Development, Homeostasis and Disease,” International Journal of Biochemistry & Cell Biology 178 (2025): 106703. 39592067 10.1016/j.biocel.2024.106703

[187] P. Heukels , C. C. Moor , J. H. von der Thüsen , M. S. Wijsenbeek , and M. Kool , “Inflammation and Immunity in IPF Pathogenesis and Treatment,” Respiratory Medicine 147 (2019): 79–91. 30704705 10.1016/j.rmed.2018.12.015

[188] J. Hou , J. Ji , X. Chen , et al., “Alveolar Epithelial Cell‐derived Sonic Hedgehog Promotes Pulmonary Fibrosis through OPN‐dependent Alternative Macrophage Activation,” The FEBS Journal 288 (2021): 3530–3546. 33314622 10.1111/febs.15669

[189] X. Yang , Z. Liu , J. Zhou , et al., “SPP1 promotes the Polarization of M2 Macrophages through the Jak2/Stat3 Signaling Pathway and Accelerates the Progression of Idiopathic Pulmonary Fibrosis,” International Journal of Molecular Medicine 54 (2024): 89. 39129313 10.3892/ijmm.2024.5413 PMC11335352

[190] G. Li , F. Jin , J. Du , Q. He , B. Yang , and P. Luo , “Macrophage‐secreted TSLP and MMP9 Promote Bleomycin‐induced Pulmonary Fibrosis,” Toxicology and Applied Pharmacology 366 (2019): 10–16. 30653976 10.1016/j.taap.2019.01.011

[191] P. W. Noble , “Epithelial Fibroblast Triggering and Interactions in Pulmonary Fibrosis,” European Respiratory Review 17 (2008): 123–129.

[192] T. Kadota , Y. Yoshioka , Y. Fujita , et al., “Extracellular Vesicles from Fibroblasts Induce Epithelial‐Cell Senescence in Pulmonary Fibrosis,” American Journal of Respiratory Cell and Molecular Biology 63 (2020): 623–636. 32730709 10.1165/rcmb.2020-0002OC

[193] F. Salton , M. Volpe , and M. Confalonieri , “Epithelial–Mesenchymal Transition in the Pathogenesis of Idiopathic Pulmonary Fibrosis,” Medicina 55 (2019): 83. 30925805 10.3390/medicina55040083 PMC6524028

[194] N. Sakai and A. M. Tager , “Fibrosis of Two: Epithelial Cell‐fibroblast Interactions in Pulmonary Fibrosis,” Biochim Biophys Acta BBA—Mol Basis Dis 1832 (2013): 911–921. 10.1016/j.bbadis.2013.03.001 PMC4041487 23499992

[195] X. Xu , J. Zhang , and H. Dai , “IL‐25/IL‐33/TSLP Contributes to Idiopathic Pulmonary Fibrosis: Do Alveolar Epithelial Cells and (myo)Fibroblasts Matter?,” Experimental Biology and Medicine (Maywood NJ) 245 (2020): 897–901. 10.1177/1535370220915428 PMC7268928 32249602

[196] B. C. Willis , J. M. Liebler , K. Luby‐Phelps , et al., “Induction of Epithelial‐mesenchymal Transition in Alveolar Epithelial Cells by Transforming Growth Factor‐beta1: Potential Role in Idiopathic Pulmonary Fibrosis,” American Journal of Pathology 166, no. 5 (2005): 1321–1332. 15855634 10.1016/s0002-9440(10)62351-6 PMC1606388

[197] A. Robles‐Remacho , R. M. Sanchez‐Martin , and J. J. Diaz‐Mochon , “Spatial Transcriptomics: Emerging Technologies in Tissue Gene Expression Profiling,” Analytical Chemistry 95 (2023): 15450–15460. 37814884 10.1021/acs.analchem.3c02029 PMC10603609

[198] L. P. M. H. de Rooij , L. M. Becker , L. A. Teuwen , et al., “The Pulmonary Vasculature in Lethal COVID‐19 and Idiopathic Pulmonary Fibrosis at Single‐cell Resolution,” Cardiovascular Research 119, no. 2 (2023): 520–535. 35998078 10.1093/cvr/cvac139 PMC9452154

[199] L. Franzén , M. Olsson Lindvall , M. Hühn , et al., “Mapping Spatially Resolved Transcriptomes in human and Mouse Pulmonary Fibrosis,” Nature Genetics 56 (2024): 1725–1736. 38951642 10.1038/s41588-024-01819-2 PMC11319205

[200] Z. Yang , G. Cao , X. Tan , et al., “Distinct Mural Cells and Fibroblasts Promote Pathogenic Plasma Cell Accumulation in Idiopathic Pulmonary Fibrosis,” European Respiratory Journal 65 (2025): 2401114. 39978854 10.1183/13993003.01114-2024 PMC12140907

[201] X. Yang , Z. Liu , J. Zhou , et al., “SPP1 promotes the Polarization of M2 Macrophages through the Jak2/Stat3 Signaling Pathway and Accelerates the Progression of Idiopathic Pulmonary Fibrosis,” International Journal of Molecular Medicine 54 (2024): 89. 39129313 10.3892/ijmm.2024.5413 PMC11335352

[202] C. Meng , G. Fan , J. Liu , N. Tao , and T. Sun , “Pirfenidone and nintedanib Exert Additive Antifibrotic Effects by the SPP1‐AKT Pathway in Macrophages and Fibroblasts,” Biochemical and Biophysical Research Communications 716 (2024): 150020. 38692011 10.1016/j.bbrc.2024.150020

[203] Y. Koda , M. Itoh , and S. Tohda , “Effects of MERTK Inhibitors UNC569 and UNC1062 on the Growth of Acute Myeloid Leukaemia Cells,” Anticancer Research 38 (2018): 199–204. 29277773 10.21873/anticanres.12208

[204] J. Wu , L. N. Frady , R. E. Bash , et al., “MerTK as a Therapeutic Target in Glioblastoma,” Neuro‐Oncology 20 (2018): 92–102. 28605477 10.1093/neuonc/nox111 PMC5761530

[205] J. H. Yi , J. Jang , J. Cho , et al., “MerTK Is a Novel Therapeutic Target in Gastric Cancer,” Oncotarget 8 (2017): 96656–96667. 29228560 10.18632/oncotarget.3750 PMC5722512

[206] E. P. Manning , A. Losier , N. Emeagwali , C. Ryu , and S. Honiden , “New Applications of Old Drugs as Novel Therapies in Idiopathic Pulmonary Fibrosis. Metformin, Hydroxychloroquine, and Thyroid Hormone,” American Journal of Respiratory and Critical Care Medicine 199 (2019): 1561–1563. 30822095 10.1164/rccm.201809-1700RR PMC7051474

[207] R. Roskoski , “Src Protein‐tyrosine Kinase Structure, Mechanism, and Small Molecule Inhibitors,” Pharmacological Research 94 (2015): 9–25. 25662515 10.1016/j.phrs.2015.01.003

[208] S. Saito , M. Kitabatake , N. Ouji‐Sageshima , et al., “Angiopoietin‐Like 4 Is a Critical Regulator of Fibroblasts during Pulmonary Fibrosis Development,” American Journal of Respiratory Cell and Molecular Biology 69 (2023): 328–339. 37192434 10.1165/rcmb.2022-0304OC

[209] P. Montero , J. Milara , I. Roger , and J. Cortijo , “Role of JAK/STAT in Interstitial Lung Diseases; Molecular and Cellular Mechanisms,” International Journal of Molecular Sciences 22 (2021): 6211. 34207510 10.3390/ijms22126211 PMC8226626

[210] X. Zhang , J. Su , J. Lin , et al., “Fu‐Zheng‐Tong‐Luo Formula Promotes Autophagy and Alleviates Idiopathic Pulmonary Fibrosis by Controlling the Janus Kinase 2/Signal Transducer and Activator of Transcription 3 Pathway,” Journal of Ethnopharmacology 314 (2023): 116633. 37207878 10.1016/j.jep.2023.116633

[211] J. Hou , T. Ma , H. Cao , et al., “TNF‐α‐induced NF‐κB Activation Promotes Myofibroblast Differentiation of LR‐MSCs and Exacerbates Bleomycin‐induced Pulmonary Fibrosis,” Journal of Cellular Physiology 233 (2018): 2409–2419. 28731277 10.1002/jcp.26112

[212] L. T. Krug , E. Torres‐González , Q. Qin , et al., “Inhibition of NF‐kappaB Signaling Reduces Virus Load and Gammaherpesvirus‐induced Pulmonary Fibrosis,” American Journal of Pathology 177 (2010): 608–621. 20566741 10.2353/ajpath.2010.091122 PMC2913377

[213] X. Li , Q. Liang , S. Gao , et al., “Lenalidomide Attenuates Post‐inflammation Pulmonary Fibrosis through Blocking NF‐κB Signaling Pathway,” International Immunopharmacology 103 (2022): 108470. 34952465 10.1016/j.intimp.2021.108470

[214] K. Komura , K. Yanaba , M. Horikawa , et al., “CD19 regulates the Development of Bleomycin‐induced Pulmonary Fibrosis in a Mouse Model,” Arthritis and Rheumatism 58 (2008): 3574–3584. 18975313 10.1002/art.23995

[215] A. Yoshizaki , Y. Iwata , K. Komura , et al., “CD19 regulates Skin and Lung Fibrosis via Toll‐Like Receptor Signaling in a Model of Bleomycin‐induced Scleroderma,” American Journal of Pathology 172 (2008): 1650–1663. 18467694 10.2353/ajpath.2008.071049 PMC2408424

[216] K. Streicher , S. Sridhar , M. Kuziora , et al., “Baseline Plasma Cell Gene Signature Predicts Improvement in Systemic Sclerosis Skin Scores Following Treatment with Inebilizumab (MEDI‐551) and Correlates with Disease Activity in Systemic Lupus Erythematosus and Chronic Obstructive Pulmonary Disease,” Arthritis Rheumatol Hoboken NJ 70 (2018): 2087–2095. 10.1002/art.40656 29956883

[217] J. Milara , G. Hernandez , B. Ballester , et al., “The JAK2 Pathway Is Activated in Idiopathic Pulmonary Fibrosis,” Respiratory Research 19 (2018): 24. 29409529 10.1186/s12931-018-0728-9 PMC5801676

[218] G. Raghu , M. Mouded , D. C. Chambers , et al., “A Phase IIb Randomized Clinical Study of an Anti‐αvβ6 Monoclonal Antibody in Idiopathic Pulmonary Fibrosis,” American Journal of Respiratory and Critical Care Medicine 206 (2022): 1128–1139. 35771569 10.1164/rccm.202112-2824OC

[219] L. Lancaster , V. Cottin , M. Ramaswamy , et al., “Bexotegrast in Patients with Idiopathic Pulmonary Fibrosis: the INTEGRIS‐IPF Clinical Trial,” American Journal of Respiratory and Critical Care Medicine 210 (2024): 424–434. 38843105 10.1164/rccm.202403-0636OC PMC11351797

[220] F. Ahangari , C. Becker , D. G. Foster , et al., “Saracatinib, a Selective Src Kinase Inhibitor, Blocks Fibrotic Responses in Preclinical Models of Pulmonary Fibrosis,” American Journal of Respiratory and Critical Care Medicine 206 (2022): 1463–1479. 35998281 10.1164/rccm.202010-3832OC PMC9757097

[221] N. Khalil , T. V. Parekh , R. O'Connor , et al., “Regulation of the Effects of TGF‐beta 1 by Activation of Latent TGF‐beta 1 and Differential Expression of TGF‐beta Receptors (T beta R‐I and T beta R‐II) in Idiopathic Pulmonary Fibrosis,” Thorax 56, no. 12 (2001): 907–915. 11713352 10.1136/thorax.56.12.907 PMC1745982

[222] E. B. Reed , S. Orbeta , B. A. Miao , et al., “Anoctamin‐1 Is Induced by TGF‐β and Contributes to Lung Myofibroblast Differentiation,” American Journal of Physiology. Lung Cellular and Molecular Physiology 326 (2024): L111–L123. 38084409 10.1152/ajplung.00155.2023 PMC11279757

[223] G. Raghu , M. Mouded , A. Prasse , et al., “Randomized Phase IIa Clinical Study of an Anti‐αvβ6 Monoclonal Antibody in Idiopathic Pulmonary Fibrosis,” American Journal of Respiratory and Critical Care Medicine 206 (2022): 1166–1168. 35830489 10.1164/rccm.202205-0868LE PMC9704833

[224] M. L. Decaris , J. R. Schaub , C. Chen , et al., “Dual Inhibition of αvβ6 and αvβ1 Reduces Fibrogenesis in Lung Tissue Explants from Patients with IPF,” Respiratory Research 22 (2021): 265. 34666752 10.1186/s12931-021-01863-0 PMC8524858

[225] T. Isshiki , S. Naiel , M. Vierhout , et al., “Therapeutic Strategies to Target Connective Tissue Growth Factor in Fibrotic Lung Diseases,” Pharmacology & Therapeutics 253 (2024): 108578. 38103794 10.1016/j.pharmthera.2023.108578

[226] A. L. MacLean , T. Hong , and Q. Nie , “Exploring Intermediate Cell States through the Lens of Single Cells,” Current Opinion in Systems Biology 9 (2018): 32–41. 30450444 10.1016/j.coisb.2018.02.009 PMC6238957

[227] T. M. Yamawaki , D. R. Lu , D. C. Ellwanger , et al., “Systematic Comparison of High‐throughput Single‐cell RNA‐seq Methods for Immune Cell Profiling,” BMC Genomics [Electronic Resource] 22 (2021): 66. 33472597 10.1186/s12864-020-07358-4 PMC7818754

[228] D. Jovic , X. Liang , H. Zeng , L. Lin , F. Xu , and Y. Luo , “Single‐cell RNA Sequencing Technologies and Applications: a Brief Overview,” Clinical and Translational Medicine 12 (2022): e694. 35352511 10.1002/ctm2.694 PMC8964935

[229] D. Arceneaux , Z. Chen , A. J. Simmons , et al., “A Contamination Focused Approach for Optimizing the Single‐cell RNA‐seq Experiment,” iScience 26 (2023): 107242. 37496679 10.1016/j.isci.2023.107242 PMC10366499

[230] H. Chung , C. N. Parkhurst , E. M. Magee , et al., “Joint Single‐cell Measurements of Nuclear Proteins and RNA in Vivo,” Nature Methods 18 (2021): 1204–1212. 34608310 10.1038/s41592-021-01278-1 PMC8532076

[231] A. Vannan , R. Lyu , A. L. Williams , et al., “Spatial Transcriptomics Identifies Molecular Niche Dysregulation Associated with Distal Lung Remodeling in Pulmonary Fibrosis,” Nature Genetics 57 (2025): 647–658. 39901013 10.1038/s41588-025-02080-x PMC11906353

[232] Z. Wen , W. Liang , Z. Yang , et al., “Genetic Insights into Idiopathic Pulmonary Fibrosis: a Multi‐omics Approach to Identify Potential Therapeutic Targets,” Journal of Translational Medicine 23 (2025): 337. 40091050 10.1186/s12967-025-06368-8 PMC11912729

